# Supplementary material for: Ontogeny of the spinal cord dorsal horn
Source: bioRxiv. 2025 Mar 15:2025.03.14.643370. Preprint. [Version 1] doi: 10.1101/2025.03.14.643370 (PMC11952496; doi:10.1101/2025.03.14.643370)
Supplement: Supplement 1 [file media-1.pdf]

Supplemental material:

Figure S1: Generation of embryonic spinal cord single-cell RNA-sequencing data

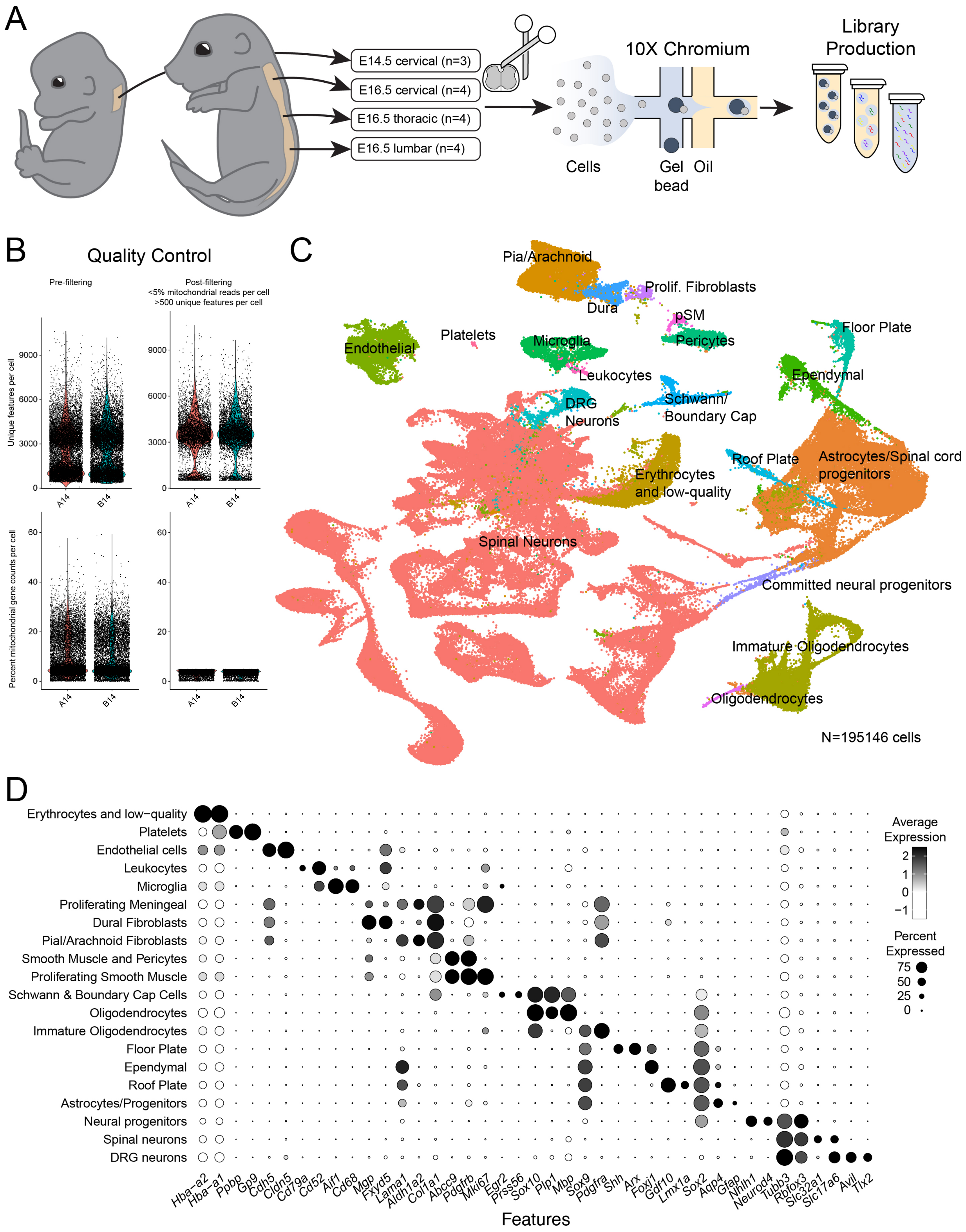

Figure S1: Generation of embryonic spinal cord single-cell RNA-sequencing data, related to Figure 1.

(A) Spinal cords were extracted from E14.5 and E16.5 spinal cords, dissociated, run through the 10X Chromium 3' gene expression pipeline, and libraries were produced and sequenced. (B) After importation into Seurat, poor quality cells with low unique features or a high percentage of mitochondrial gene reads were removed from the dataset. (C) UMAP plot of the high-quality cells, following integration and clustering. (D) Dot plot showing all major annotated spinal cord cell types, showing marker gene sets used to classify clusters into archetypal spinal cord cell types. Dot size represents the fraction of a cell type expressing a gene, and dot shading represents the average normalized expression of a gene per cell type.
Numbers: Pooled embryonic spinal cord replicates (A): E14.5 cervical spinal cord (n=3), E16.5 cervical spinal cord (n=4), E16.5 thoracic spinal cord (n=4), E16.5 lumbosacral spinal cord (n=4).
Total cells after quality control: N=195146.
Abbreviations: DEG – differentially-expressed gene, UMAP – uniform manifold approximation and projection.

Figure S2: Zic1 expression defines the boundary of dI4/dILA and dI5/dILB

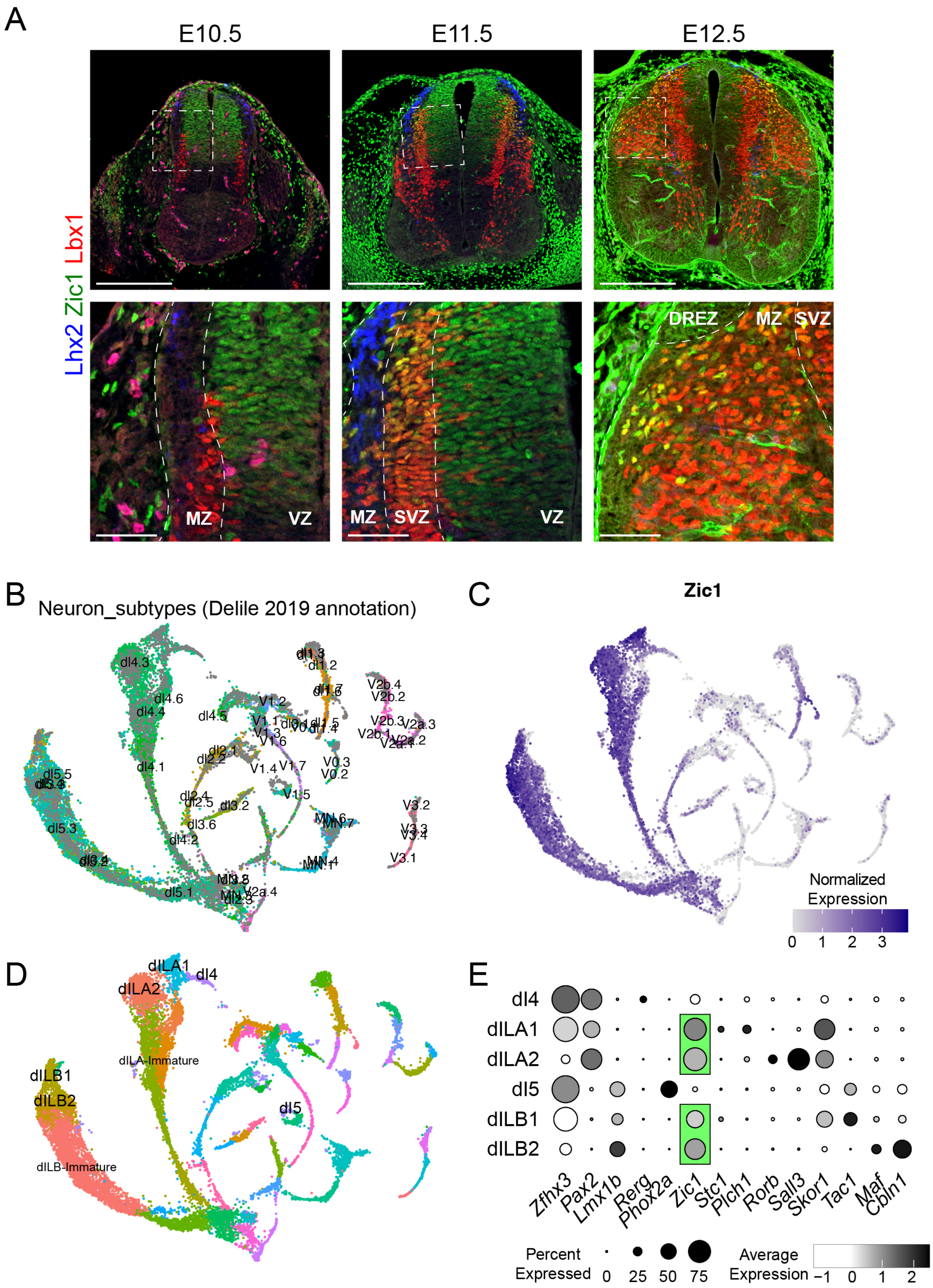

Figure S2: *Zic1* expression defines the boundary of dI4/dILA and dI5/dILB, related to Figure 1.

(A) Expression of Zic1 in embryonic day (E) 10.5, E11.5 and E12.5 spinal cords. Zic1 is expressed in dorsal progenitors throughout neurogenesis, appears in newborn dIL neurons (Lbx1+) at E11.5, and is expressed in varying degrees throughout dIL neurons at E12.5. Notably it is absent from dI4-6 neurons marked by Lbx1 at E10.5, as well as dI1 neurons marked by Lhx2.
(B-D) UMAP plots of single-cell RNA-sequencing data from E9.5 – E13.5 spinal cords from Delile et al, 2019 and Osseward et al, 2021, encompassing the timeframe of (A), showing *Zic1* expression in dIL neurons but not dI4/5 neurons. (B) Cluster annotations from Delile et al., 2019. (C) Normalized expression of *Zic1*. (D) Re-annotation of the Delile data based on our analysis. (E) Dot plot showing that the earliest-born dorsal horn neurons (dI4/dI5) by TTF expression do not express *Zic1*, while those bearing dILA1/2 and dILB1/2 markers do. Dot size represents the fraction of a cell type expressing a gene, and dot shading represents the average normalized expression of a gene per cell type.
Numbers: Representative images from n=3 spinal cords from E10.5, E11.5 and E12.5. Single-cell RNA-sequencing cells: Delile et al., 2019 (N=356 E9.5, N=1724 E10.5, N=3588 E11.5, N=4561 E12.5, N=5542 E13.5); Osseward et al., 2021 (N=2914 E12.5).
Scale bars: (A) 250 mm (top row), 50 mm (bottom row).
Abbreviations: DREZ – dorsal root entry zone, MZ – marginal zone, SVZ – subventricular zone, TTF – temporal transcription factor, UMAP – uniform manifold approximation and projection, VZ – ventricular zone.

Figure S3: Transcriptomic analysis of post-neurogenesis embryonic spinal cord neurons

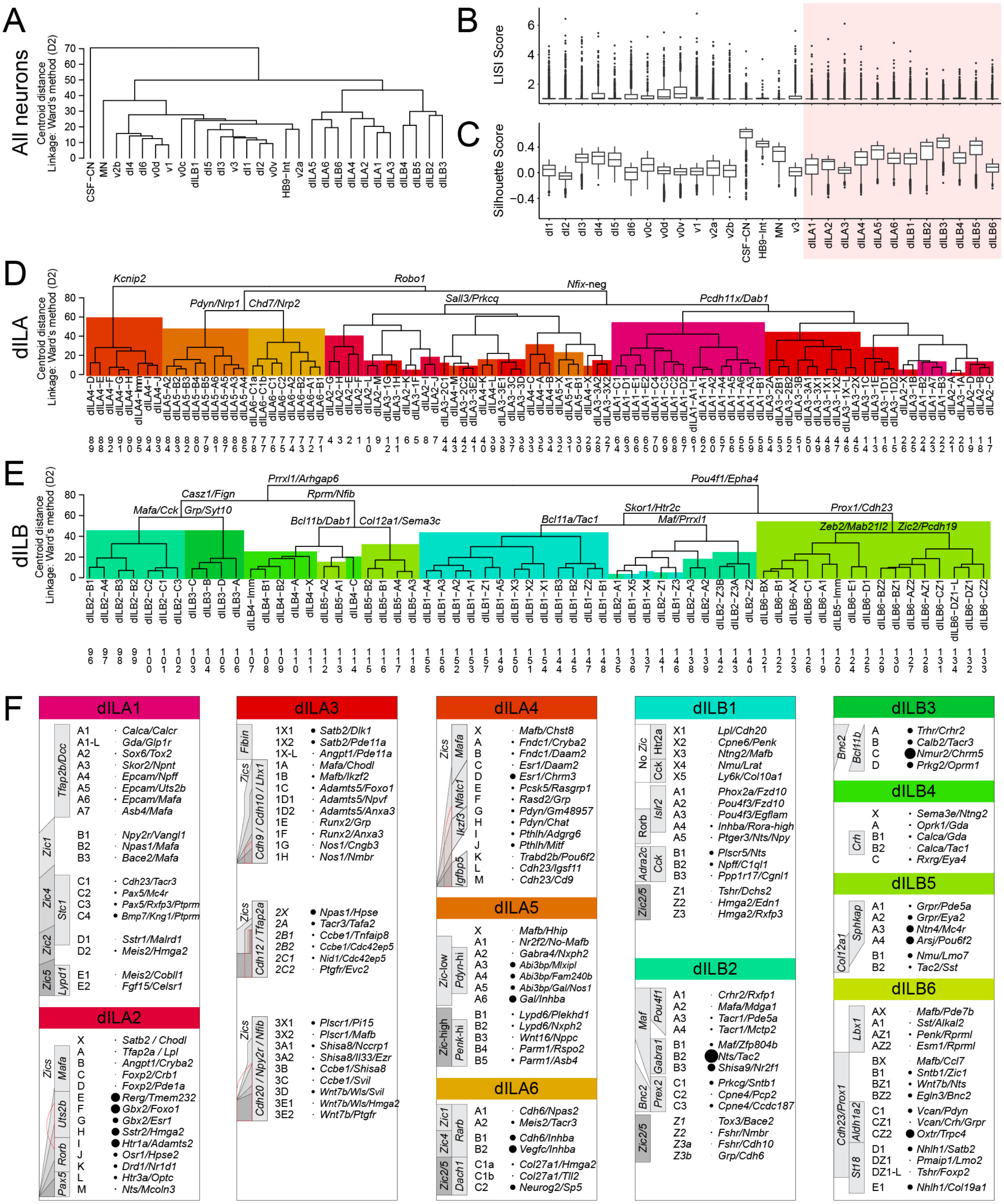

Figure S3: Transcriptomic analysis of post-neurogenesis embryonic spinal cord neurons, related to Figure 1.

A) Hierarchical relationships of all major spinal cord neuron classes by cluster centroid distance in PC space. Dendrograms were produced by Ward's method (D2), with use of the top 90 PCs. (B,C) Cluster robustness metrics for major spinal cord neuron classes, using LISI (B) and silhouette (C) scores. dIL neurons are indicated by red background. (D,E) Hierarchical relationships of dILA (D) neuron subtypes and dILB (E) neuron subtypes within each respective class by cluster centroid distance in PC space. Dendrograms were produced by Ward's method (D2), with use of 90 PCs in both cases. Colors represent major dILA and dILB families. High-order branches are annotated with top DEGs. Numbers refer to terminal dendrogram branches in Figure 1F which corresponding to subtype names indicated on terminal dendrogram branches here. F) Annotated tables for each of twelve dIL families, showing subtypes and top marker genes. The "-L" suffix represents neuron subtypes which are virtually only found samples taken from the lumbosacral cord; no outstanding neuron subtypes are exclusive to cervical or thoracic segments given the current number of dIL neurons sampled. Dot size next to subtype name corresponds to the relative frequency of dIL neuron subtype among subtypes of all six families (excluding clusters annotated as transitional or immature (those overrepresented in E14.5 data or with substantial *Robo3*/*Nhlh1* expression).
Numbers: Single-cell RNA-sequencing data analyzed here was derived from 94,048 neurons (see Figure 1C and methods for details).
Abbreviations: DEG – differentially-expressed gene, PC – principal component

Figure S4: Robustness of spinal neuron clusters using a random forest classifier

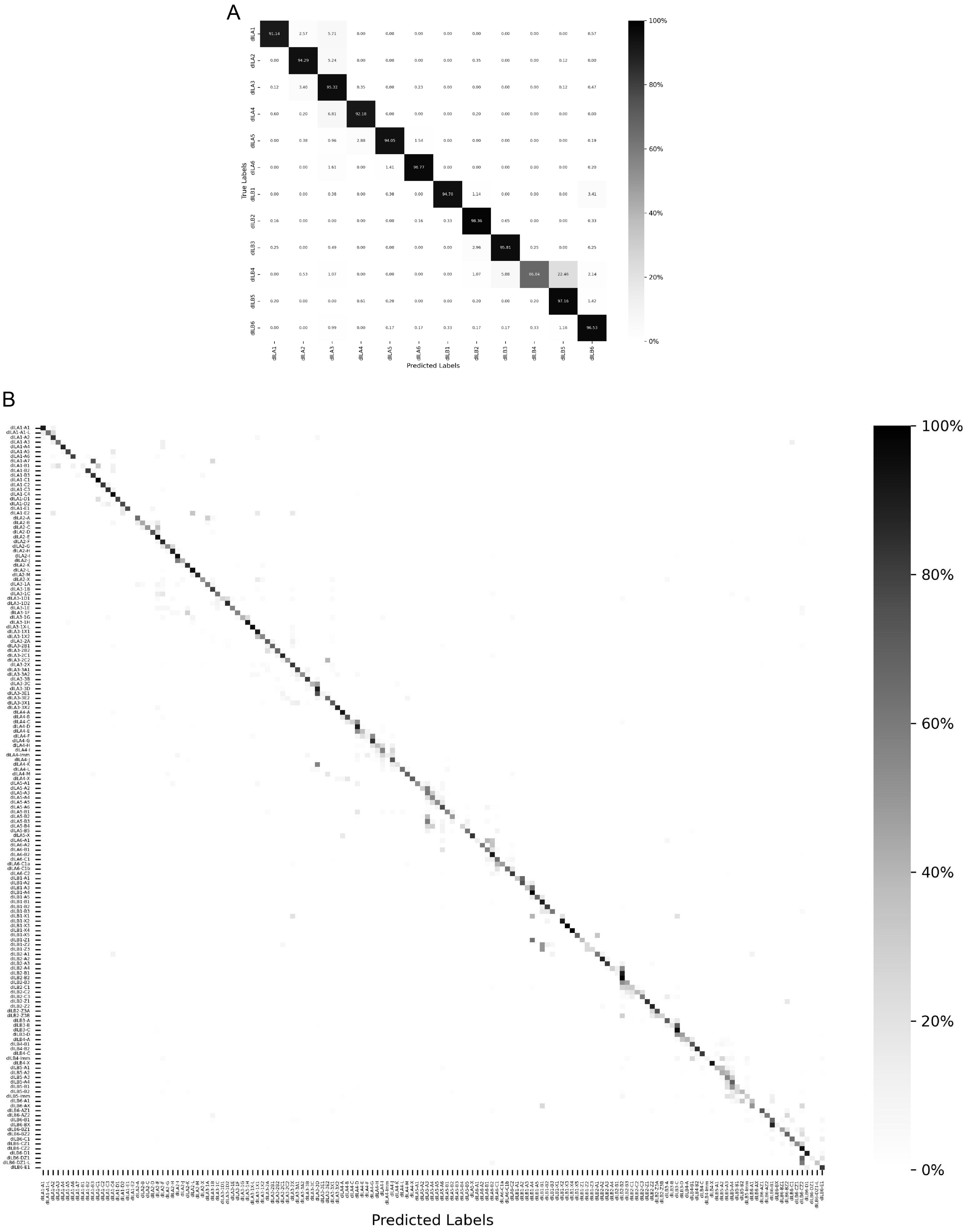

Figure S4: Robustness of spinal neuron clusters using a random forest classifier, related to Figure 1.

Confusion matrix heatmaps demonstrating the accuracy of a trained random forest classifier in predicting either family-level (A) or family subtype-level (B) annotations for individual dIL neurons using single-cell RNA-sequencing data generated and annotated here.

Figure S5: UMAP plot of dIL family subtypes

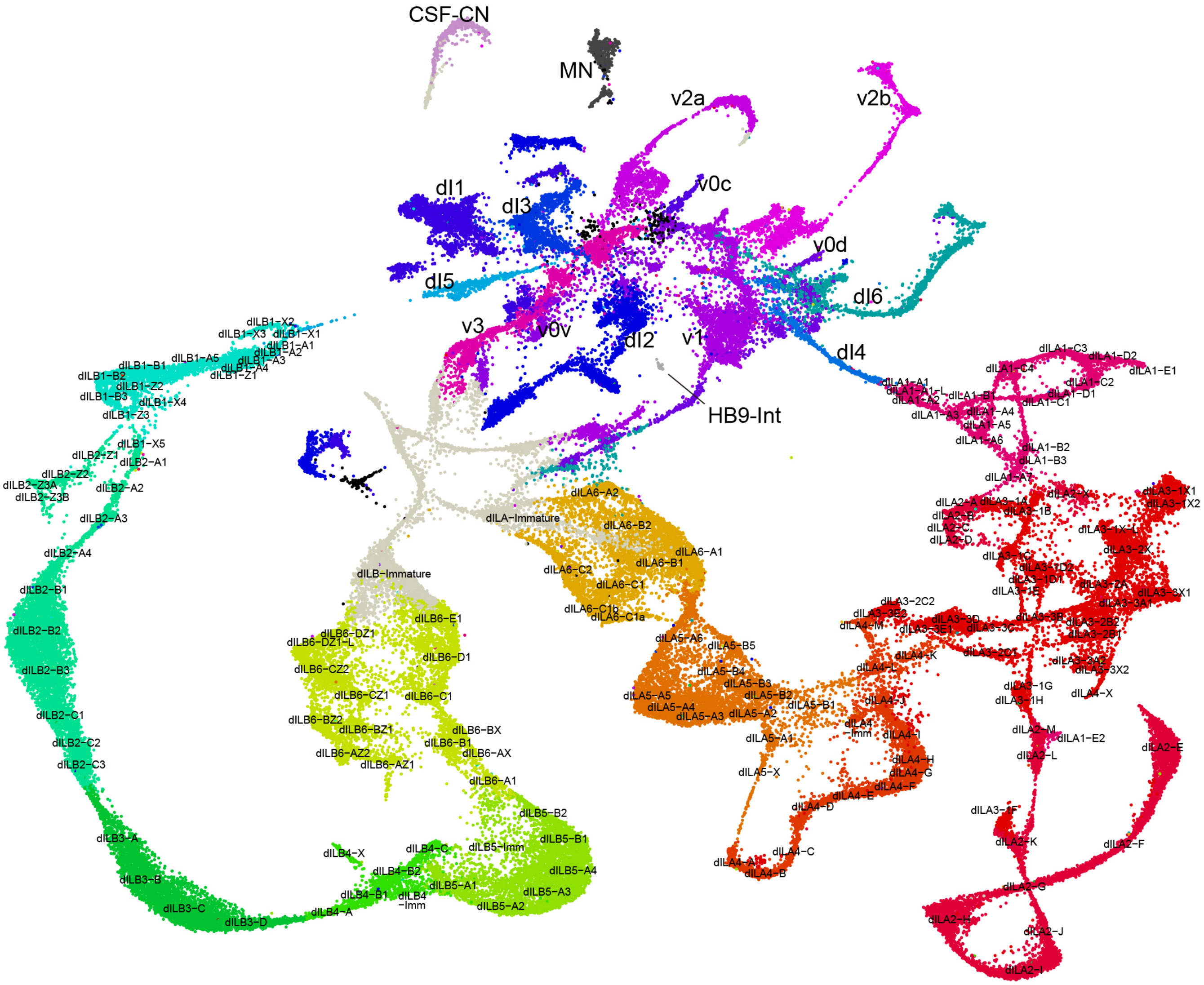

Figure S5: UMAP plot of dIL family subtypes, related to Figure 1.

UMAP plot of all spinal cord neurons, with the approximate UMAP centroid of each dIL neuron type annotated. Cardinal-class neurons are annotated in larger text.
Abbreviations: UMAP – uniform manifold approximation and projection.

Figure S6: EdU birthdating of dILA families

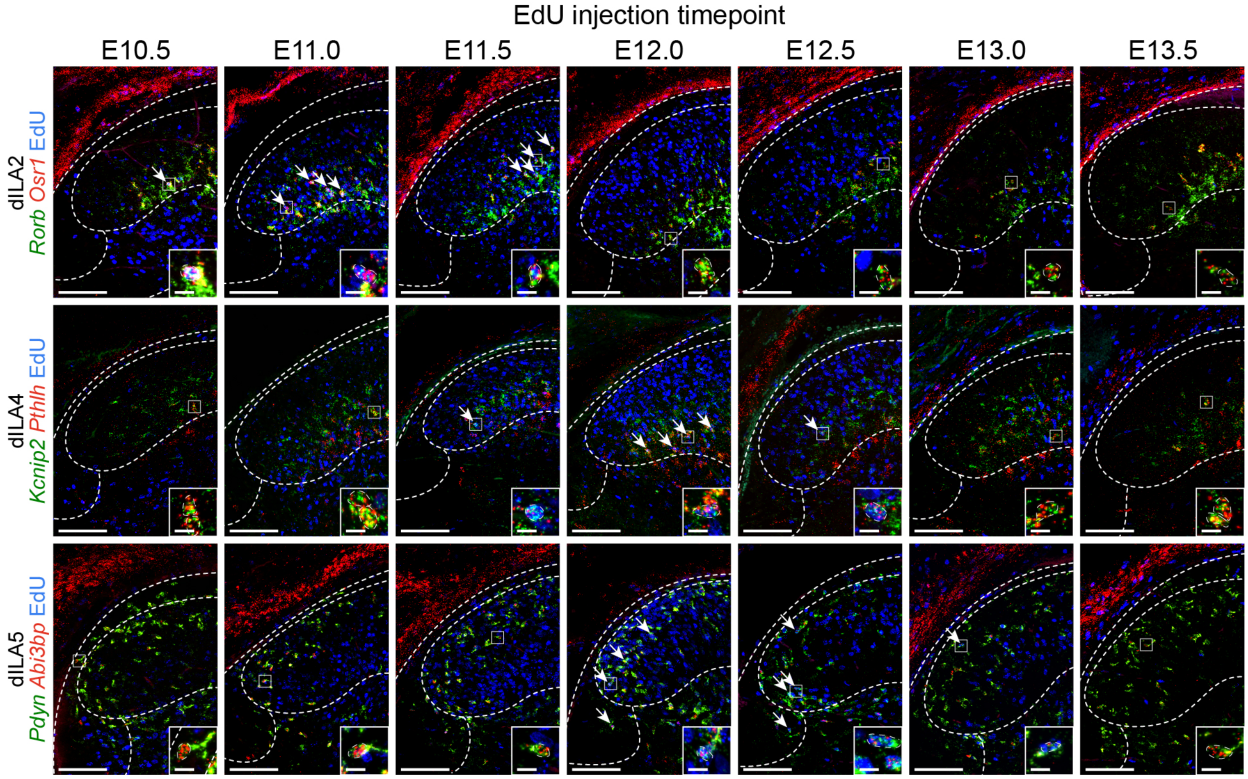

Figure S6: EdU birthdating of dILA families, related to Figure 2.

Representative images of the lateral dorsal horn of the spinal cord from E16.5 embryos having received EdU pulses at either E10.5, E11.0, E11.5, E12.0, E12.5, E13.0 or E13.5 respectively. A subset of dILA families are identified by coincident expression of two genes (red and green) revealed by RNA Scope in-situ hybridization which exclude all other dIL neuron families; dILA2 – *Rorb* and *Osr1*, dILA4 – *Kcnip2* and *Pthlh*, dILA5 – *Pdyn* and *Abi3bp*. Arrows indicate dILA family neurons expressing the corresponding marker gene set and which are labeled with nuclear EdU (blue). Dashed lines indicate the border of the superficial dorsal horn with the deep dorsal horn and the lateral spinal nucleus, the lateral spinal nucleus with the lateral funiculus, and the pia. Insets indicate examples of triple-positive neurons surrounded by dashed lines. Example images from this figure used in Figure 2E are reused here for contextual purposes.
Numbers: Individual litters of embryos from a mother given a single EdU injection were considered a single biological replicate. E10.5 (n=5 litters), E11.0 (n=4 litters), E11.5 (n=5 litters), E12.0 (n=5 litters), E12.5 (n=5 litters), E13.0 (n=5 litters), E13.5 (n=4 litters).
Scale bars: 250 μm, 25 μm for inset panels.
Abbrevations: EdU - 5-ethynyl-2'-deoxyuridine.

Figure S7: EdU birthdating of dILB families

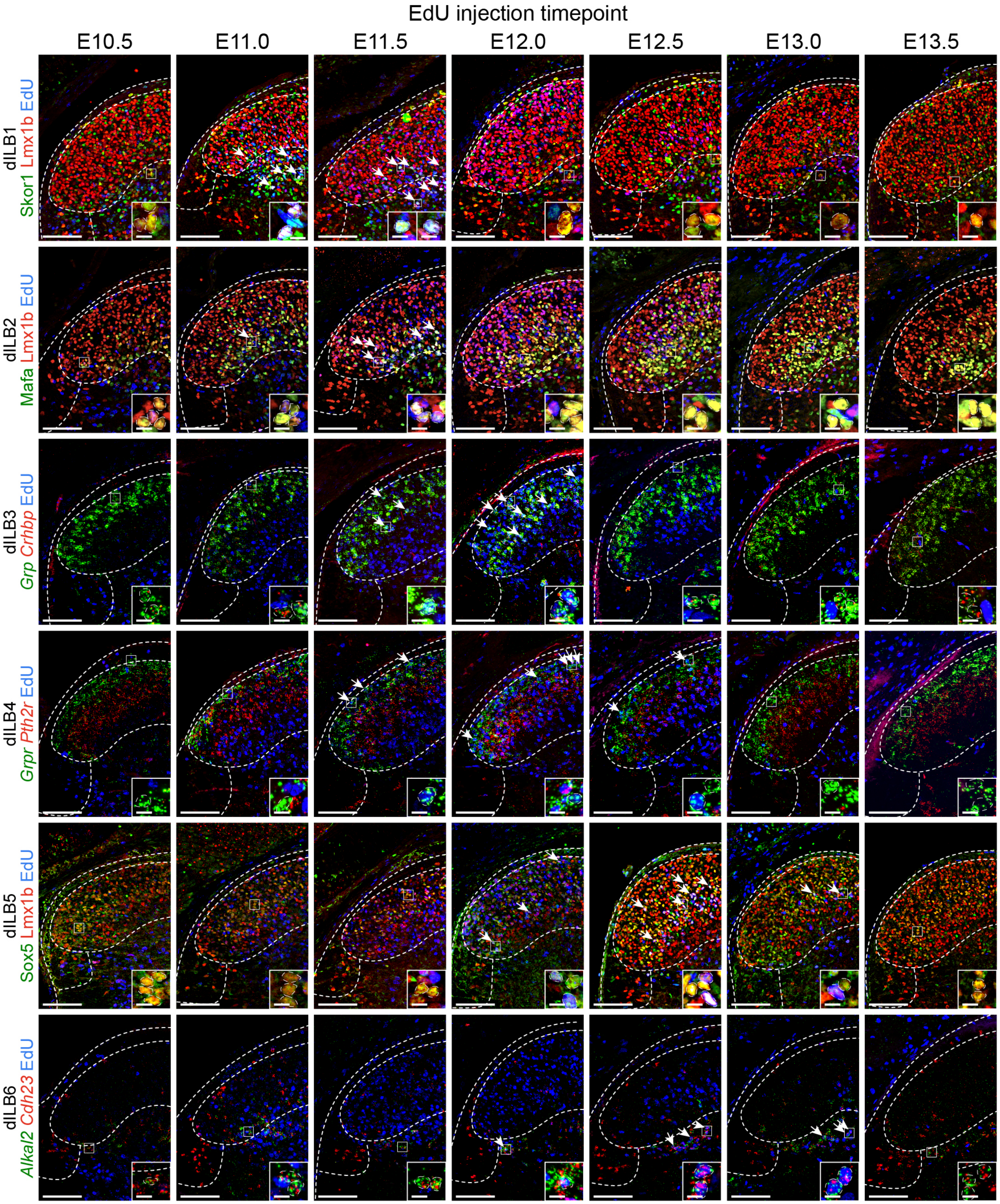

Figure S7: EdU birthdating of dILB families, related to Figure 2.

Representative images of the lateral dorsal horn of the spinal cord from E16.5 embryos having received EdU pulses at either E10.5, E11.0, E11.5, E12.0, E12.5, E13.0 or E13.5 respectively. A subset of dILB families are identified by coincident expression of two genes (red or green) revealed by RNA Scope in-situ hybridization or nuclear protein expression; dILB1 – Lmx1b and Skor1, dILB2 – Lmx1b and Mafa, dILB3 – *Grp* and *Crhbp*, dILB4 – *Grpr* excluding *Pth2r*, dILB5 – Lmx1b and Sox5, dILB6 – *Cdh23* and *Alkal2*. Arrows indicate dILB family neurons expressing the corresponding marker gene set and which are labeled with nuclear EdU (blue). Dashed lines indicate the border of the superficial dorsal horn with the deep dorsal horn and the lateral spinal nucleus, the lateral spinal nucleus with the lateral funiculus, and the pia. Insets indicate examples of triple-positive neurons surrounded by dashed lines. Example images from this figure used in Figure 2E are reused here for contextual purposes.
Numbers: Individual litters of embryos from a mother given a single EdU injection were considered a single biological replicate. E10.5 (n=5 litters), E11.0 (n=4 litters), E11.5 (n=5 litters), E12.0 (n=5 litters), E12.5 (n=5 litters), E13.0 (n=5 litters), E13.5 (n=5 litters).
Scale bars: 250 μm, 25 μm for inset panels.
Abbrevations: EdU - 5-ethynyl-2'-deoxyuridine.

Figure S8: EdU birthdating of dILB neurons in a cohort unpaired with in-situ hybridization

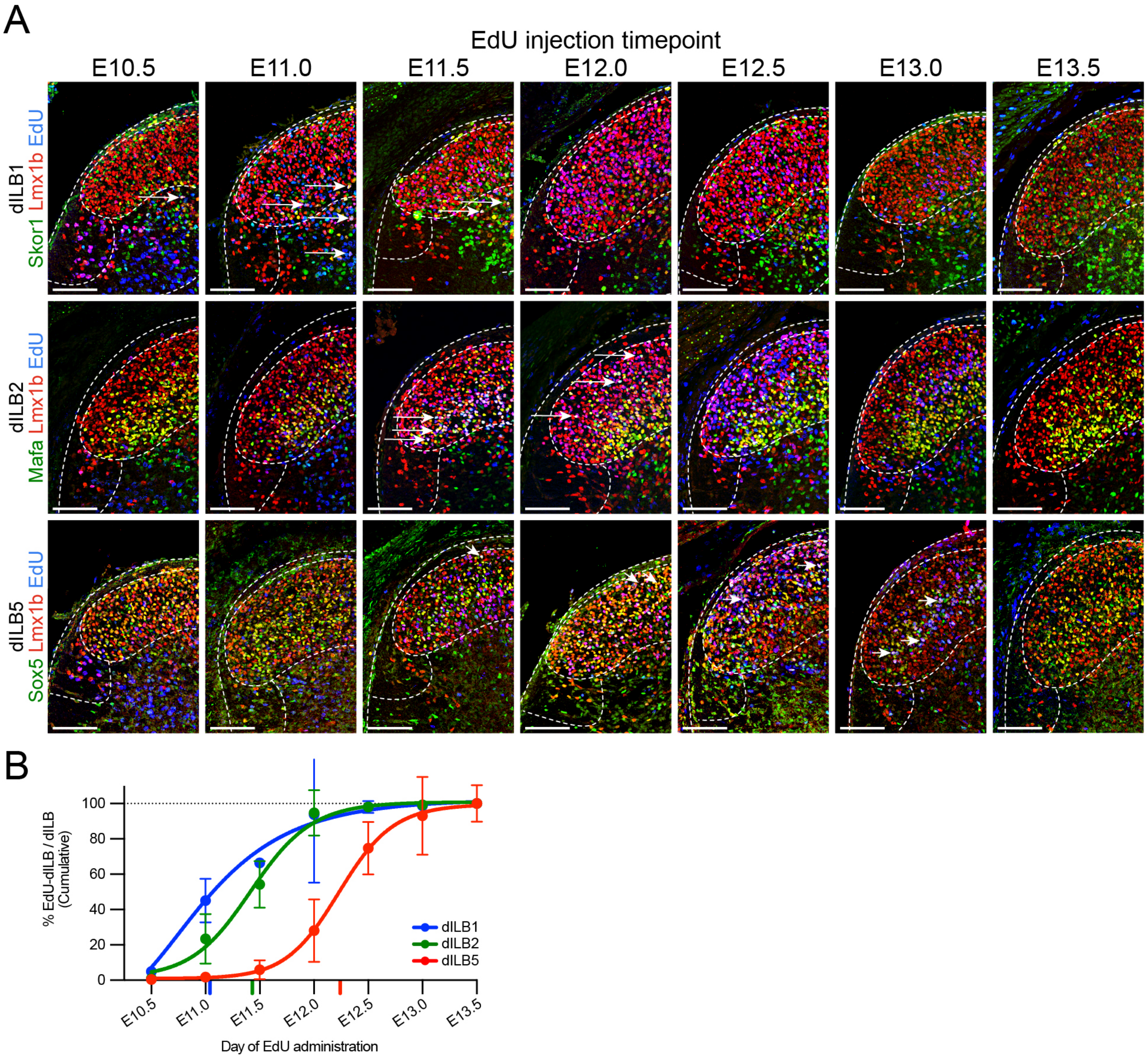

Figure S8: EdU birthdating of dILB neurons in a cohort unpaired with in-situ hybridization, related to Figure 2.

Embryos from birthdated litters where separate embryos were not appropriately fixed for in-situ hybridization were only examined for dILB families identifiable using immunohistochemistry (dILB1, dILB2 and dILB5). This experiment was supplanted by those in Figures 2, S7 and S8, and thus not all litters were collected in triplicate prior to conducting subsequent experiments. Despite this, these experiments provide independent support the same birth order for dILB1, dILB2 and dILB5.
(A) Representative images of the lateral dorsal horn of the spinal cord from E16.5 embryos having received EdU pulses at either E10.5, E11.0, E11.5, E12.0, E12.5, E13.0 or E13.5 respectively. A subset of dILB families are identified by coincident expression of two nuclear proteins (red and green) which exclude all other dIL neuron families; dILB1 – Lmx1b and Skor1, dILB2 – Lmx1b and Mafa, dILB5 – Lmx1b and Sox5. Arrows indicate dILB family neurons expressing the corresponding marker gene set and which are labeled with nuclear EdU (blue). Dashed lines indicate the border of the superficial dorsal horn with the deep dorsal horn and the lateral spinal nucleus, the lateral spinal nucleus with the lateral funiculus, and the pia. Insets indicate examples of triple-positive neurons surrounded by dashed lines.
(B) Plot showing the cumulative labeling of dILB1, dILB2 or dILB5 neurons by EdU over subsequent embryonic days. Points represent cumulative EdU labeled for each dILB family; individual data points are mean cumulative EdU labeling for all embryos of a single litter. Lines represent best-fit sigmoidal relationships of means of each dILB family.
Numbers: Images and cell counts shown here are derived from the same cohort of embryos analyzed in Figures 1A, 1B, 3A and 3B; however, here data from all embryos in a single litter given a single EdU injection were averaged and considered a single biological replicate. E10.5 (n=2-3 litters), E11.0 (n=3 litters), E11.5 (n=4 litters), E12.0 (n=3 litters), E12.5 (n=4 litters), E13.0 (n=3 litters), E13.5 (n=4 litters).
Scale bars: 250 µm.
Abbrevations: EdU - 5-ethynyl-2'-deoxyuridine.

Figure S9: Spatial transcriptomics reveal the laminar position of dIL family subtypes

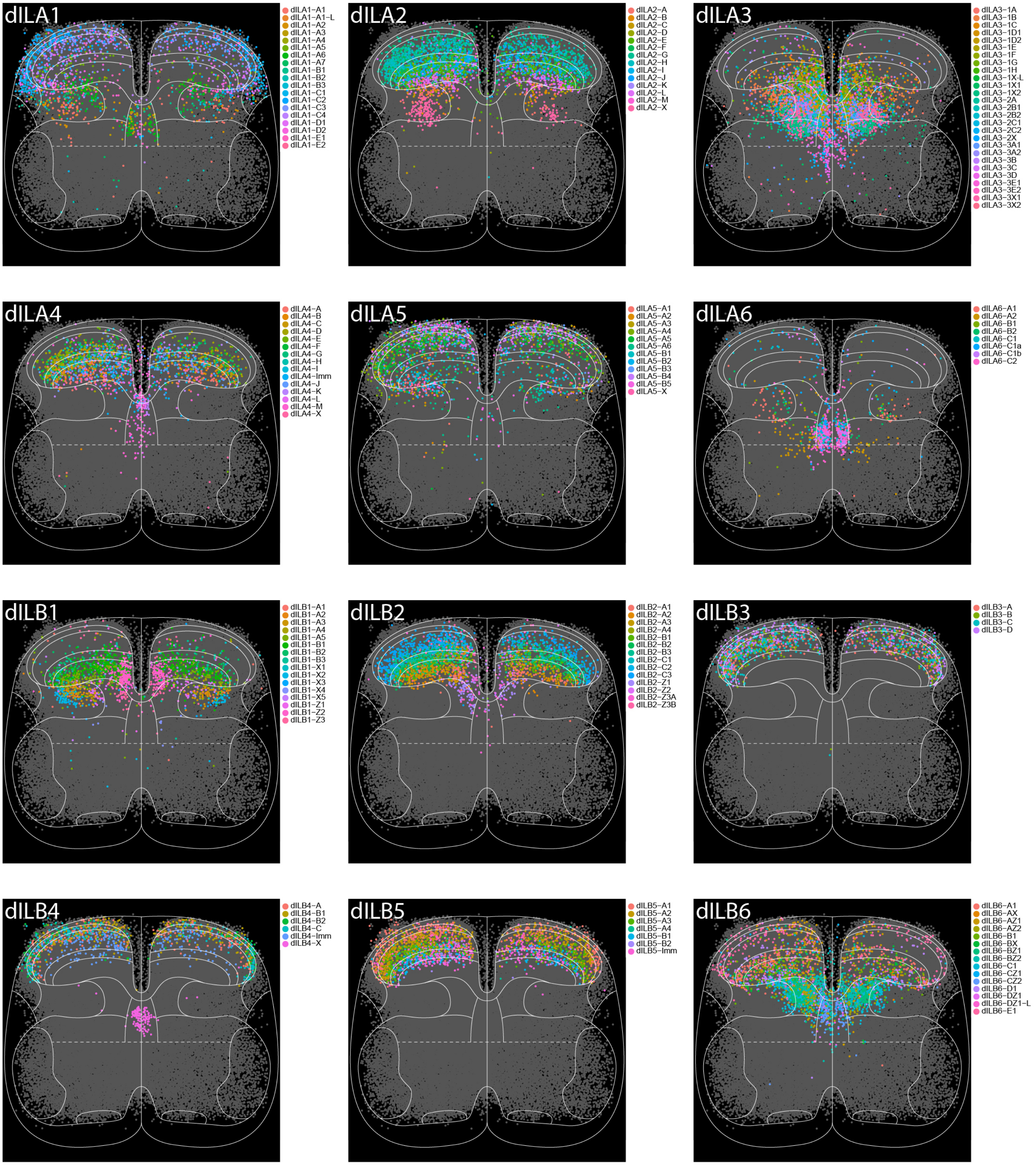

Figure S9: Spatial transcriptomics reveal the laminar position of dIL family subtypes, related to Figure 3.

Scatter plots depict the coordinates of all wild-type brachial (C5-C8) E16.5 neurons examined in the Xenium spatial transcriptomics assay. Cell-type identity was predicted using RCTD, with single-cell RNA-sequencing data from Figure 1 as a reference. Cell coordinates were normalized to anatomic landmarks from each profiled spinal cord section; subsequently, the plane of cell coordinates was rotated and stretched to fit an ideal diagram of a spinal cord (white solid lines in each panel, with approximate laminar boundaries in dashed white lines). This process was identical for all cells regardless of annotation. Cells assigned to each of 6 dILA and 6 dILB families are plotted separately, as indicated in the top left of each panel. Cells not belonging to the indicated dIL family are plotted in gray; otherwise, they are colored based on their family subtype identity in the corresponding legend to each panel.
Numbers: Cell coordinates were derived from six whole sections and seven hemisections (N=9.5 sections) among n=3 replicate wild-type E16.5 spinal cords. From these sections 54,166 cells were identified as neurons and plotted, of which 27,714 were annotated as dIL neurons.
Abbreviations: RCTD – Robust cell-type decomposition (R library).

Figure S10: dILB neurons laminate normally in absence of dILA neurons – images

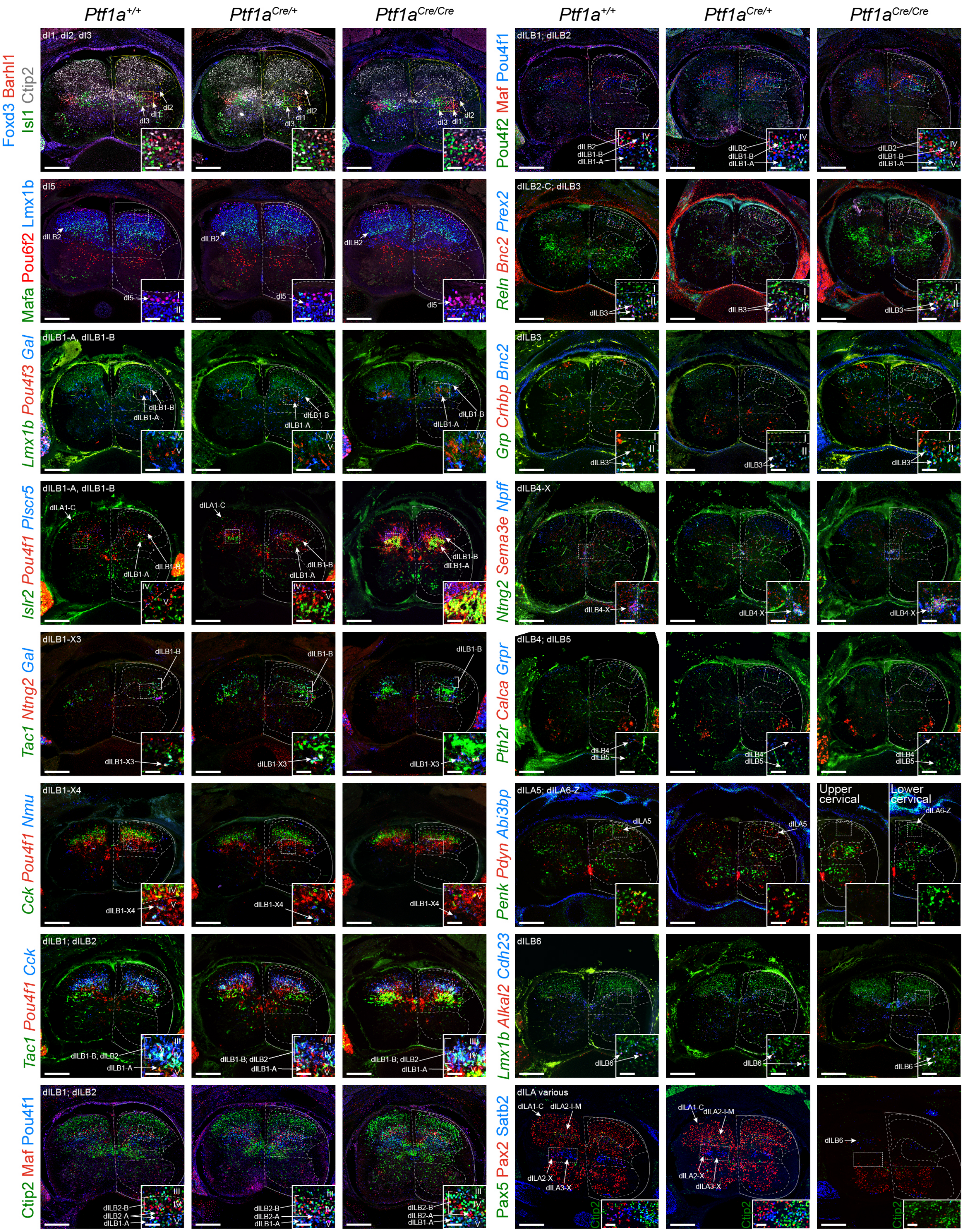

Figure S10: dILB neurons laminate normally in absence of dILA neurons – images, related to Figure 4.

Images of immunohistochemistry and RNA Scope in-situ hybridization of various cardinal excitatory, dILB dorsal horn neuron types and selected dILA populations (indicated in the top left of each row) are examined in E16.5 spinal cords from embryos wild-type, heterozygous-null, or null for *Ptf1a*. dILA neurons are largely absent from null spinal cords while excitatory neurons are anatomically similar to controls (though present in increased numbers). White arrows indicate examples of dIL family subtypes. Insets at the bottom right of each panel are magnified fields of view from boxes marked by dashed white lines in the larger panel. Other solid and dashed white lines indicate approximate anatomical boundaries of the spinal cord. Images of *Plscr5* (row 4, left) are reused here from Figure 4, for contextual purposes.
Numbers: Representatives images from n=4 *Ptf1a*$^{+/+}$; n=4 *Ptf1a*$^{Cre/+}$; n=4 *Ptf1a*$^{Cre/Cre}$ E16.5 spinal cords.
Scale bars: 250 µm, 50 µm for inset panels.

Figure S11: Transdifferentiation of dIL neurons in *Ptf1a*- and *Gsx1/2*-null embryos

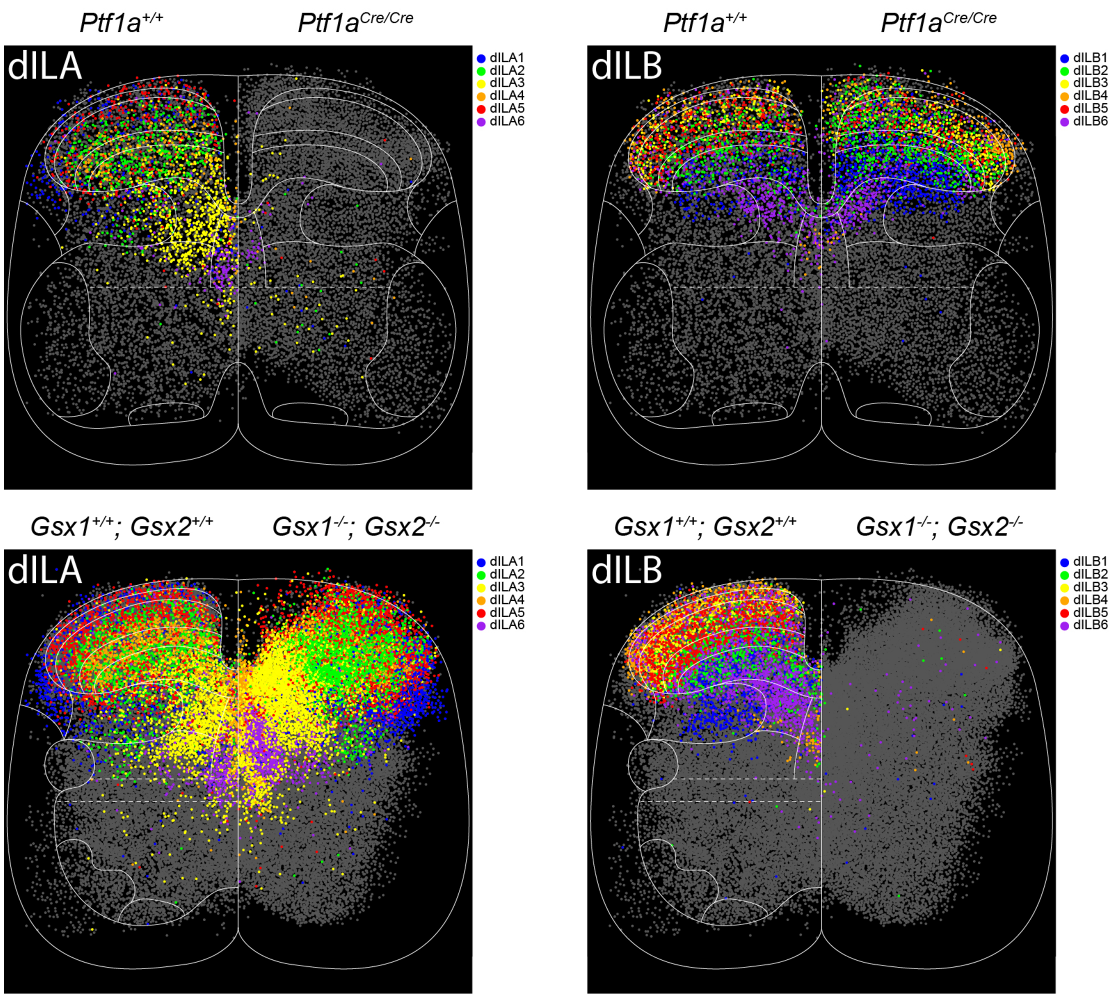

Figure S11: Transdifferentiation of dIL neurons in *Ptf1a*- and *Gsx1/2*-null embryos, related to Figure 4.

Scatter plots depict the coordinates of wild-type, *Ptf1a*-null or *Gsx1/2*-null E16.5 neurons examined in the Xenium spatial transcriptomics assay, colored by either dILA family (column 1) or dILB family (column 2). Here, segmentally-matched wild-type cell coordinates have been mirrored and overlaid on the left side of each diagram and segmentally corresponding cell coordinates from either *Ptf1a*-null tissue (row 1) or *Gsx1/2*-null tissue (row 2) have been mirrored and overlaid on the right side of each diagram in order to facilitate anatomical comparisons between genotype. *Ptf1a*-null cells have been compared to brachial wild-type cell coordinates displayed in Figure S9 while *Gsx1/2*-null tissue analyzed from upper thoracic segments have been compared to wild-type thoracic cell coordinates not previously displayed. Wild-type neurons have been evenly downsampled from each neuron subtype annotation to match the total number of null cells displayed per diagram. Notably, dILA neurons are absent from Ptf1a-null tissue and dILB neurons are absent from *Gsx1/2*-null tissue. Cells not belonging to the indicated dIL family are plotted in gray. Solid and dashed white lines indicate approximate anatomical boundaries of the spinal cord.

Numbers: Spatial transcriptomics from E16.5 wild-type embryo brachial segments was previously described (Figure S9). For E16.5 wild-type thoracic segments, cell coordinates were derived from five whole sections and one hemisection (N=5.5 sections), among n=3 replicate spinal cords. From these sections 27,758 cells were identified as neurons and plotted, of which 13,724 were annotated as dIL neurons. For E16.5 *Ptf1a*-null brachial segments, cell coordinates were derived from two whole sections (N=2 sections) among n=1 spinal cord. From these sections, 8,267 cells were identified as neurons, and of which 2,767 were dIL neurons. E16.5 brachial data was downsampled to 8,106 neurons of which 4,151 were dIL neurons to match. For E16.5 *Gsx1/2*-null thoracic segments, cell coordinates were derived from four whole sections and three hemisections (N=5.5 sections) among n=3 replicate spinal cords. From these sections, 24,737 cells were identified as neurons, and of which 12,217 were dIL neurons. E16.5 thoracic data was downsampled to 24,583 neurons with, also, 12,217 dIL neurons, to match.

Figure S12: dILB neurons laminate normally in absence of dILA neurons – Xenium

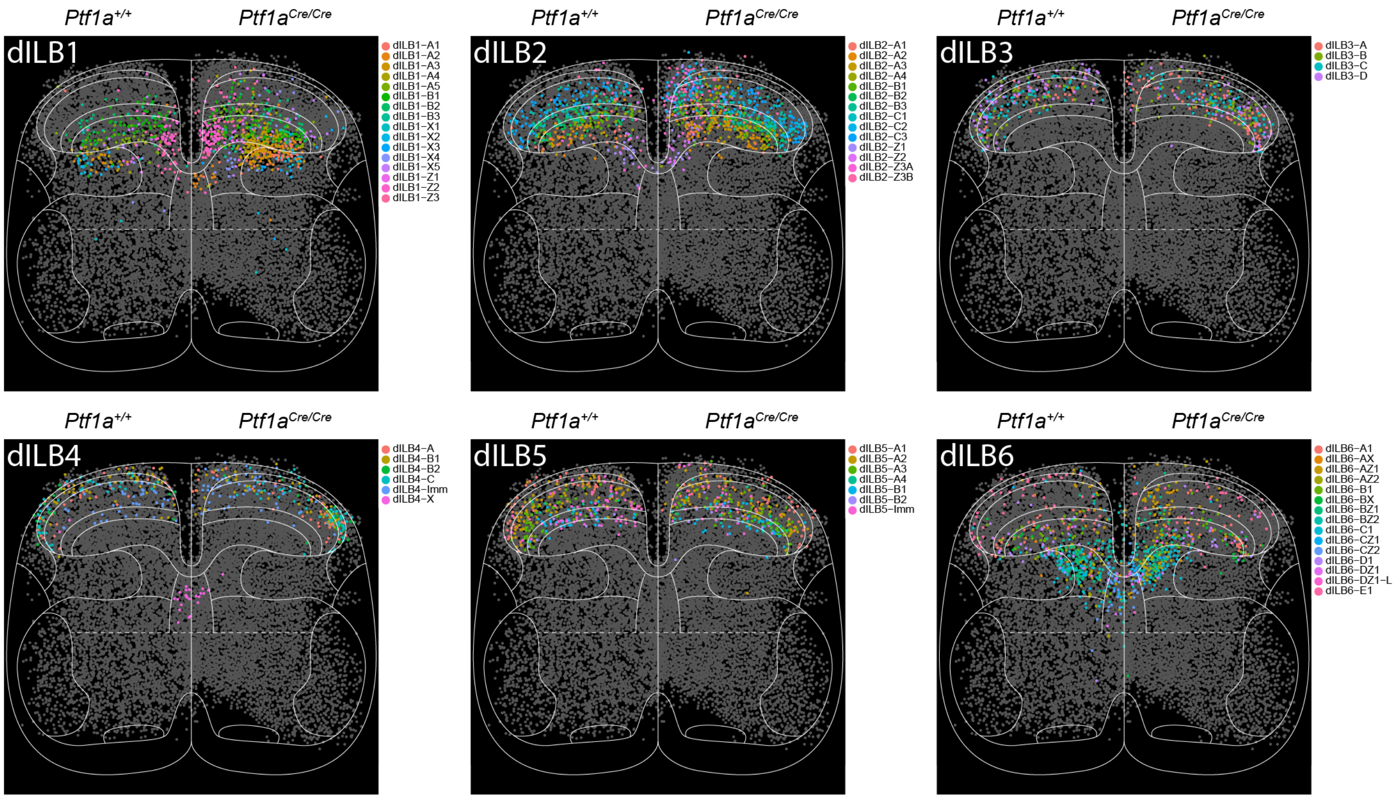

Figure S12: dILB neurons laminate normally in absence of dILA neurons – Xenium, related to Figure 4.

Scatter plots depict the coordinates of wild-type and *Ptf1a*-null E16.5 neurons examined in the Xenium spatial transcriptomics assay, sorted into each of six dILB families and colored by subtype annotation. As described previously, wild-type cells are displayed on the left and null cells on the right of each diagram, and wild-type cells were downsampled to match the number of null cells. All cells presented here are also presented in Figure 4 and S11 with family annotations. Cells not belonging to the indicated dIL family are plotted in gray. Solid and dashed white lines indicate approximate anatomical boundaries of the spinal cord.

Figure S13: dILA neurons lose laminar patterning in absence of dILB neurons – images

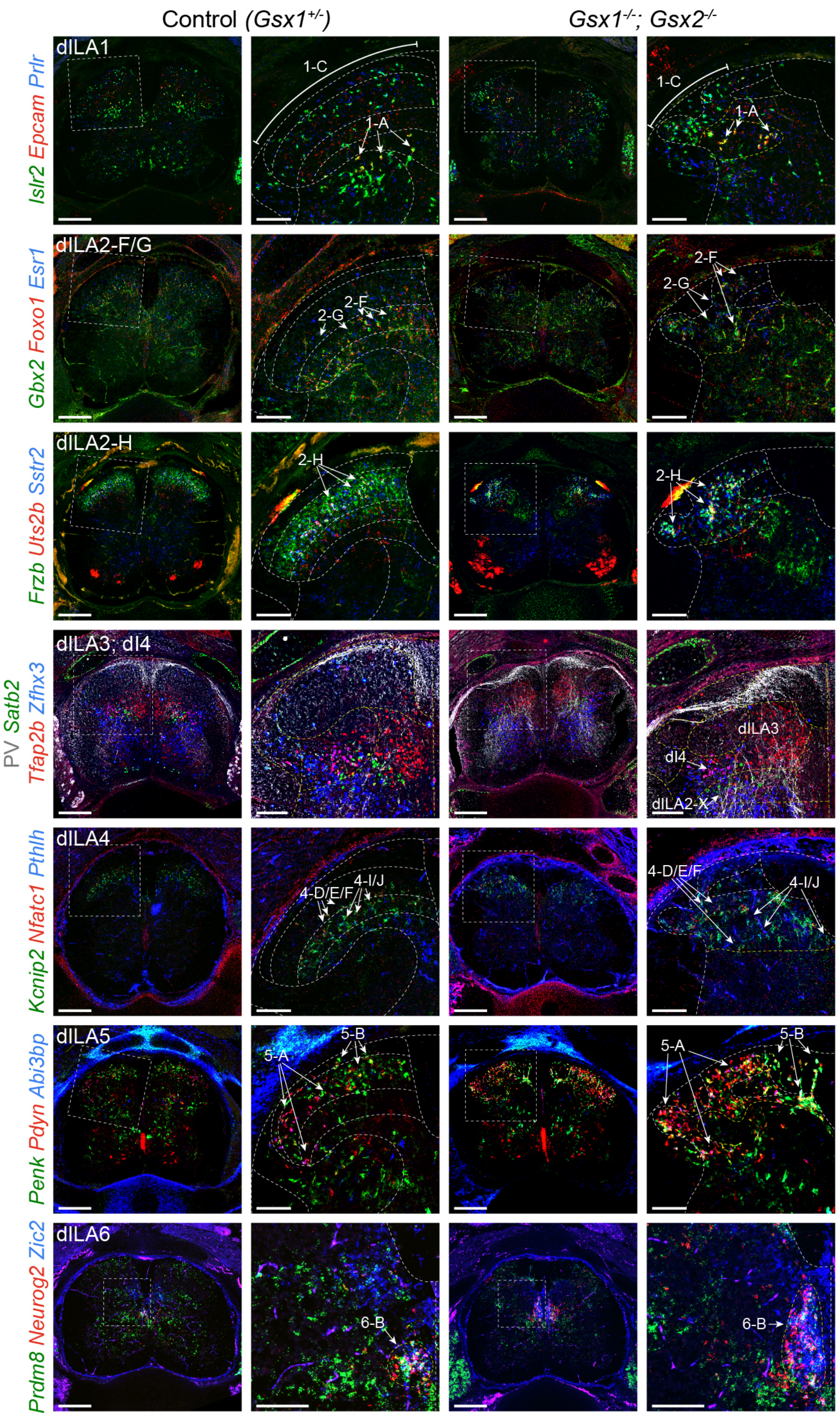

Figure S13: dILA neurons lose laminar patterning in absence of dILB neurons – images, related to Figure 4.

Images of immunohistochemistry and RNA Scope in-situ hybridization of dILA family subtypes (indicated in the top left of each row) are examined in E16.5 spinal cords from embryos heterozygous-null for *Gsx1* (as control), or null for both *Gsx1* and *Gsx2*. dILA neurons normally present in laminae are no longer present in laminae and are largely disorganized. Unlaminated dILA neurons in the deep dorsal horn are slightly displaced, though in anatomically similar locations. dILB neurons are largely absent from null spinal cords. White arrows indicate examples of dIL family subtypes. For each genotype, the left column of images depicts an entire spinal cord section while the right column depicts a magnified image from the dashed white line box in the left column. Other solid and dashed white lines indicate approximate anatomical boundaries of the spinal cord.
Numbers: Representative images from n=4 $Gsx1^{+/-}$; $Gsx2^{+/+}$, n=4 $Gsx1^{-/-}$; $Gsx2^{-/-}$.
Scale bars: 250 μm for left column, 100 μm for magnified panels (right column) for each genotype.
Images of PV (row 4) are reused here from Figure 4, for contextual purposes.

Figure S14: dILA neurons lose laminar patterning in absence of dILB neurons – Xenium

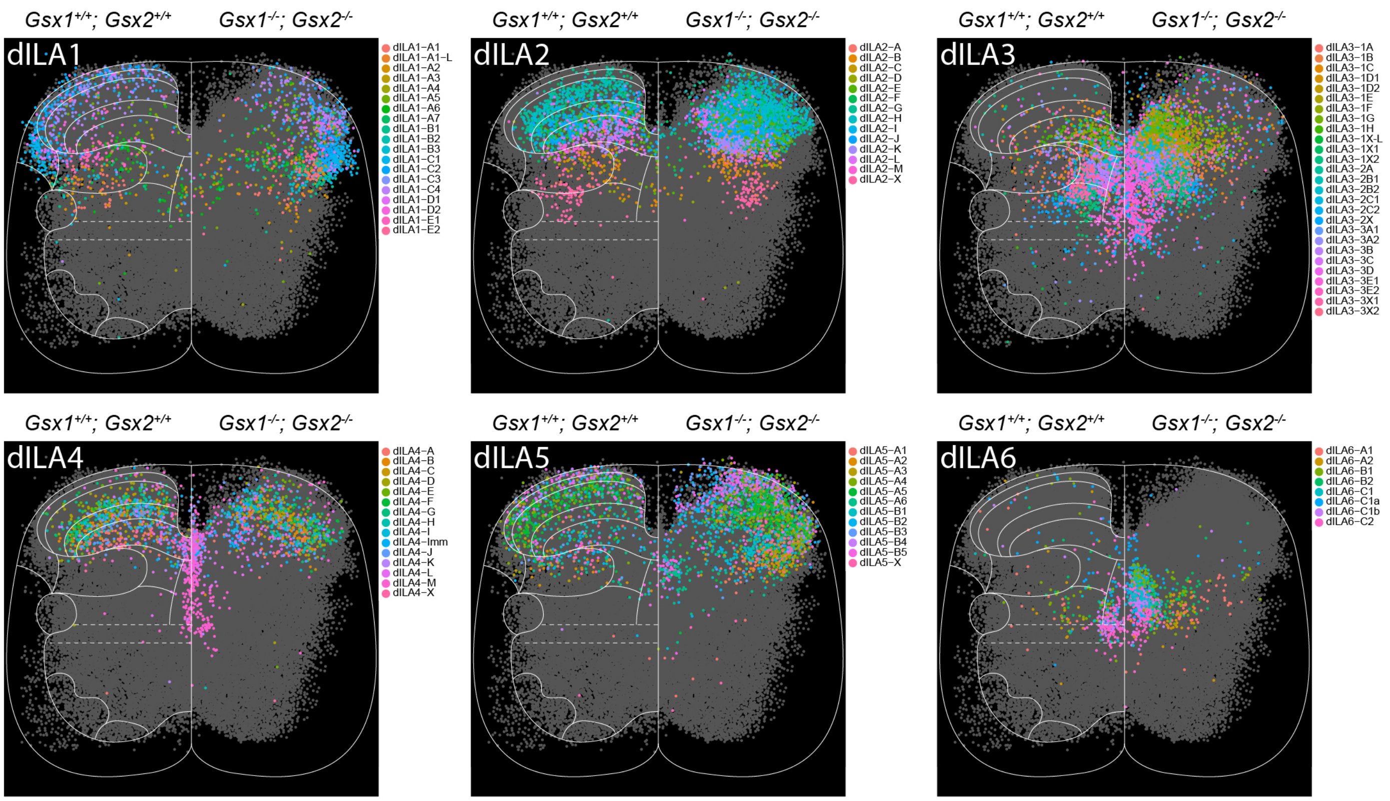

Figure S14: dILA neurons lose laminar patterning in absence of dILB neurons – Xenium, related to Figure 4.

Scatter plots depict the coordinates of wild-type and *Gsx1/2*-null E16.5 neurons examined in the Xenium spatial transcriptomics assay, sorted into each of six dILA families, and colored by subtype annotation. As described previously, wild-type cells are displayed on the left and null cells on the right of each diagram, and wild-type cells were downsampled to match the number of null cells. All cells presented here are also presented with family-level annotations in Figure S11 and with selected refined annotations in Figure 4. Cells not belonging to the indicated dIL family are plotted in gray. Solid and dashed white lines indicate approximate anatomical boundaries of the spinal cord.

Figure S15: Sensory neuron afferents are dispensable for dorsal horn structure

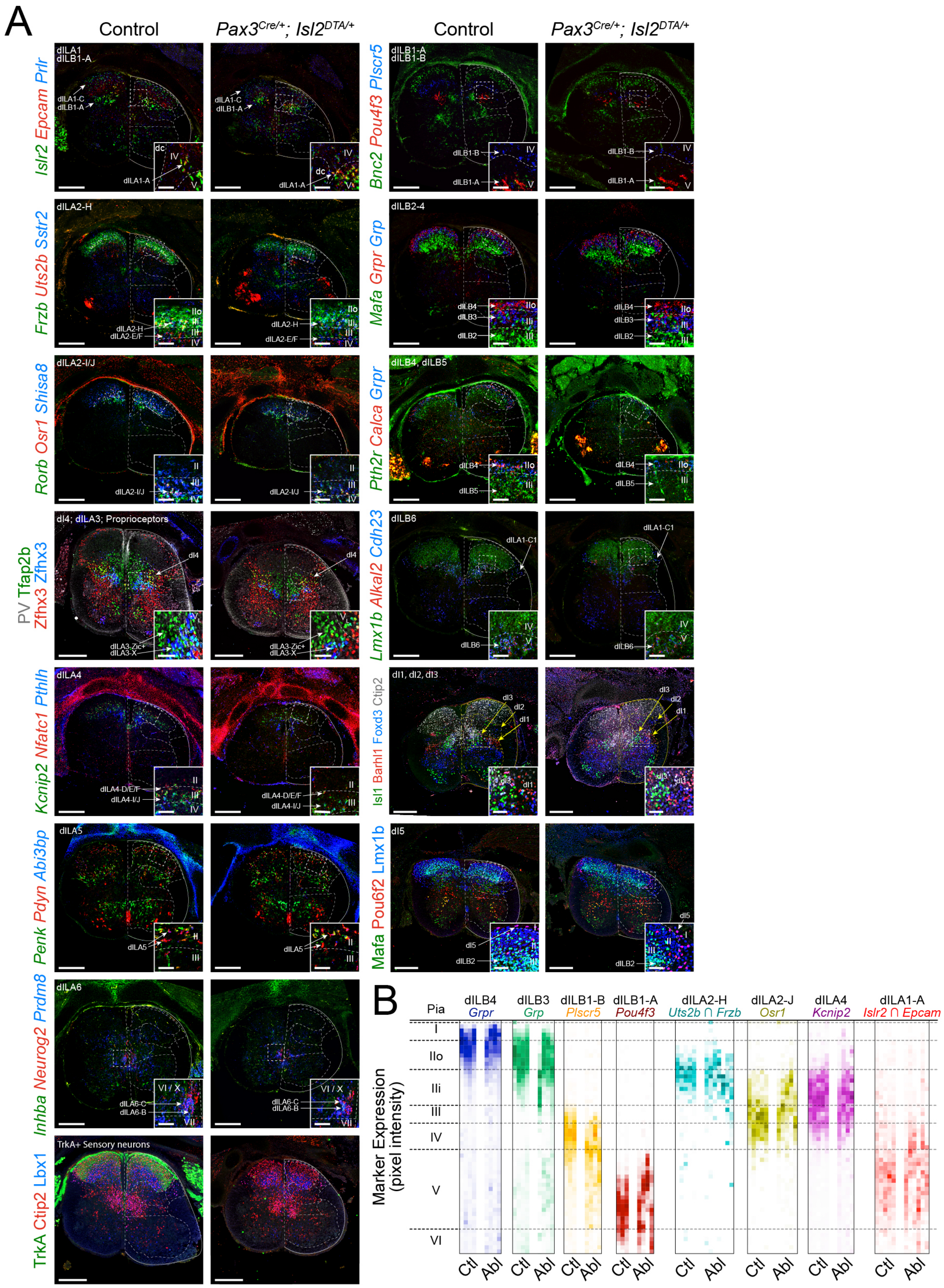

Figure S15: Sensory neuron afferents are dispensable for dorsal horn structure, related to Figure 4.

(A) Immunohistochemistry and RNA Scope in-situ hybridization of dILA family subtypes, dILB family subtypes, and sensory afferents (indicated in the top left of each row) are examined in E16.5 spinal cords from control littermates, (either *Pax3*$^{Cre/+}$ or *Isl2*$^{DTA/+}$) or embryos which are both *Pax3*$^{Cre/+}$ and *Isl2*$^{DTA/+}$, resulting in pan-sensory neuron ablation. DILA and dILB neurons laminate in sensory neuron-ablated spinal cord similarly to control spinal cords. Notable phenotypes are atrophy of the dorsal columns (due to a lack of sensory neuron axons), and mild atrophy of dorsal horn interneurons non-specifically when sensory neurons are absent. White arrows indicate examples of dIL family subtypes. Insets at the bottom right of each panel are magnified fields of view from boxes marked by dashed white lines in the larger panel. Other solid and dashed white lines indicate approximate anatomical boundaries of the spinal cord.
(B) Spatial distribution of individual gene or protein markers or marker sets (as indicated at the top of each box), showing the normalized proportion of total signal per dorso-ventral bin (color intensity) where the pia is set as the dorsal boundary and approximate laminar positions are shown. Biological replicates are grouped together as individual columns for either Ctl (control – either *Pax3*$^{Cre/+}$ or *Isl2*$^{DTA/+}$) or Abl (Ablated - *Pax3*$^{Cre/+}$; *Isl2*$^{DTA/+}$).
Numbers: Representative images and data from n=3-5 control (either *Pax3*$^{Cre/+}$ or *Isl2*$^{DTA/+}$), n=4-7 *Pax3*$^{Cre/+}$;*Isl2*$^{DTA/+}$ E16.5 spinal cords.
Scale bars: 250 µm, 50 µm for inset panels.

Figure S16: Expression of Zic family genes in the dorsal horn

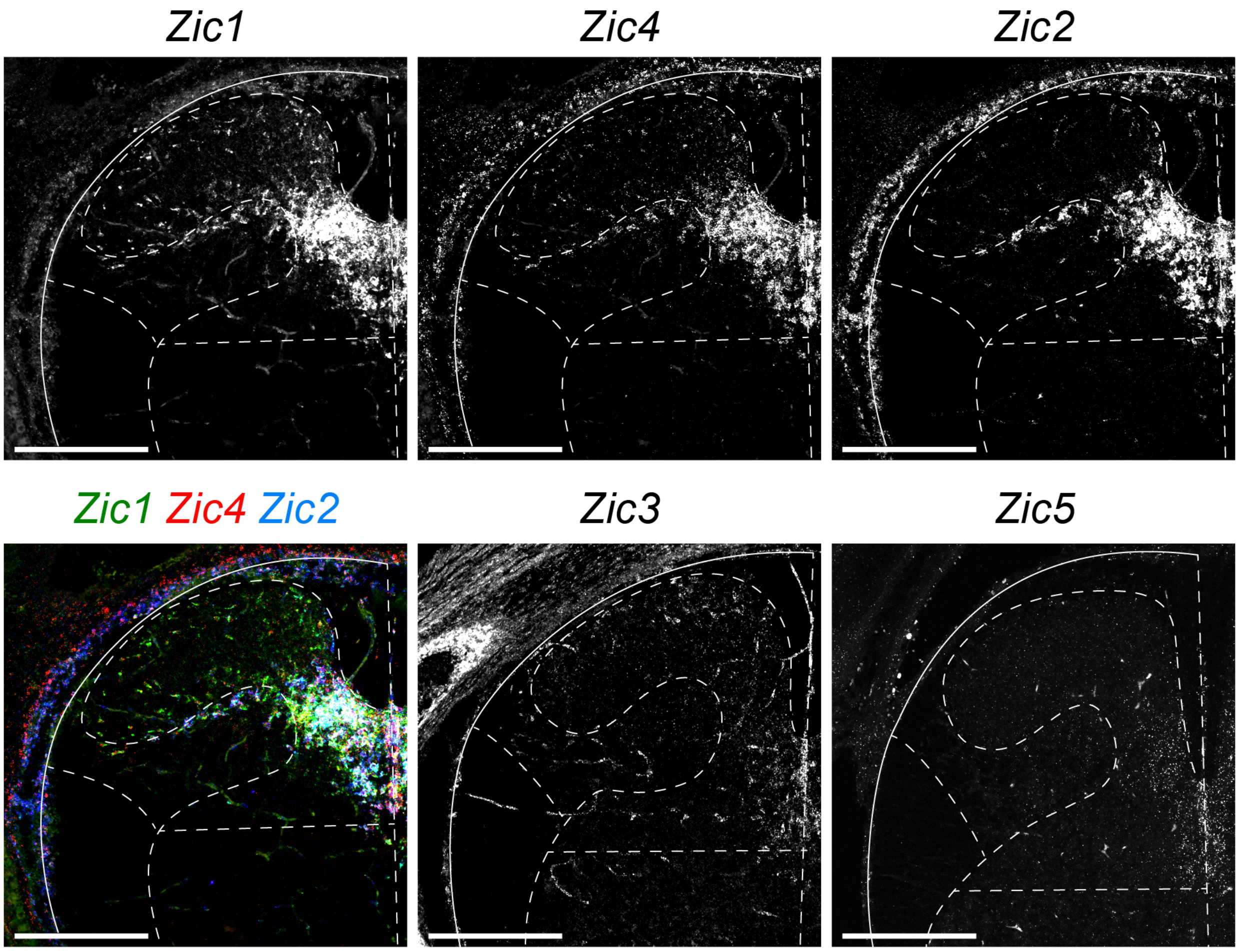

Figure S16: Expression of Zic family genes in the dorsal horn, related to Figure 5.

Zic1-5 genes are expression in stereotypical patterns restricted to dorsal horn neurons, with notable expression also along vasculature and meninges. The breadth and intensity of Zic gene expression corresponds well to that observed within single-cell RNA-sequencing of dIL neurons.

Numbers: Representative images from n=3 E16.5 wild-type mouse spinal cords

Scale bars: 250 μm

Figure S17: *Zic3* mildly represses formation of mid Zic-gradient subtypes of dIL neurons

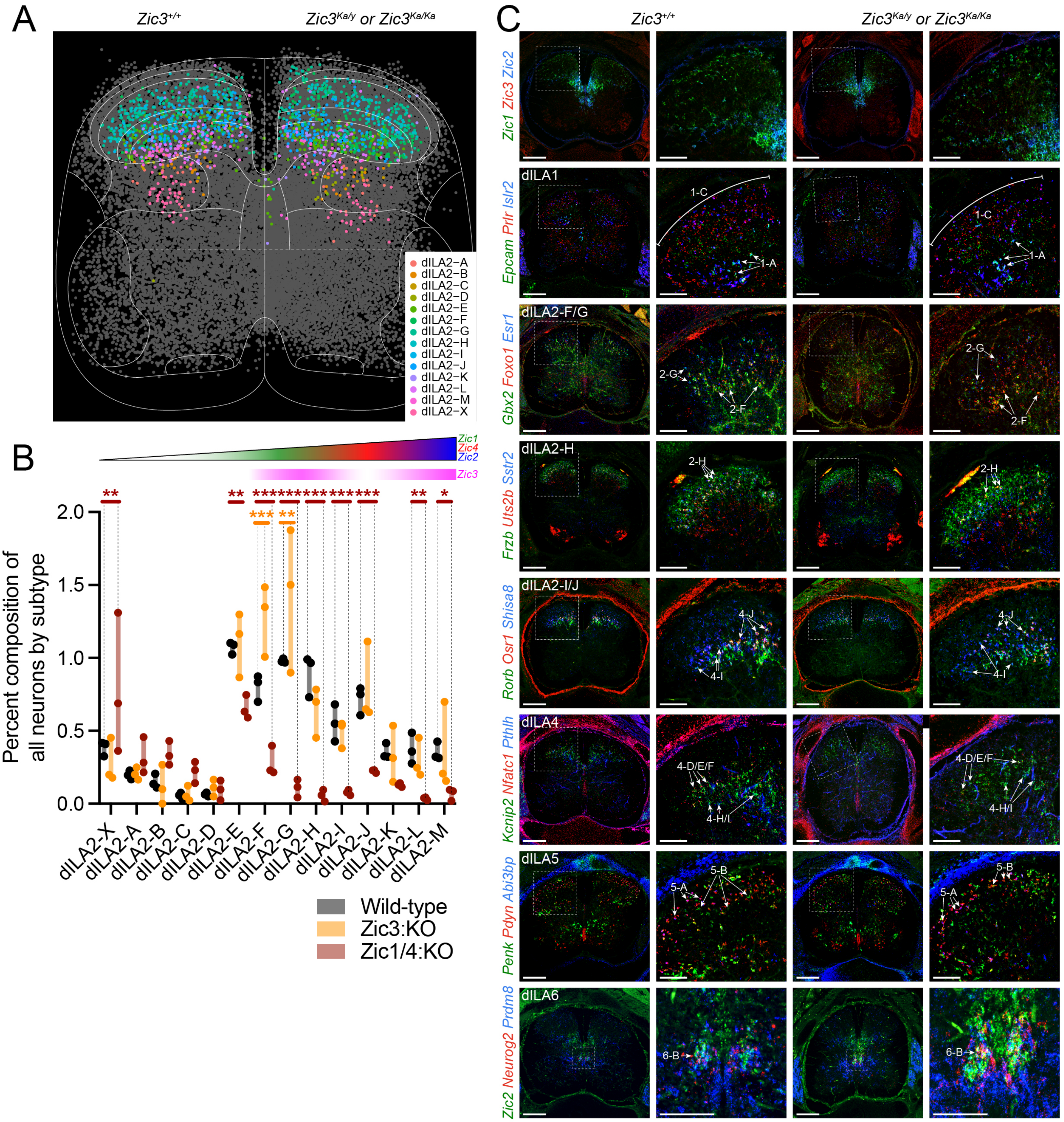

Figure S17: *Zic3* mildly represses formation of mid Zic-gradient subtypes of dIL neurons, related to Figure 5.

(A) The scatter plot depicts the coordinates of brachial wild-type and *Zic3*-null E16.5 neurons examined in the Xenium spatial transcriptomics assay and colored by subtype annotation of the dILA2 family (as an example). As described previously, wild-type cells are displayed on the left and null cells on the right of each diagram, and wild-type cells were downsampled to match the number of null cells. Cells not belonging to the dILA2 family are plotted in gray. Solid and dashed white lines indicate approximate anatomical boundaries of the spinal cord. (B) The fraction of dILA2 family subtypes of all neurons in *Zic3*-null spinal cords is depicted here compared to wild-type and *Zic1/4*-null data, gathered from predicted neuron subtype identities in Xenium spatial transcriptomics analyses. Wild-type and *Zic1/4*-null data are depicted in Figure 5K, and are displayed here again for reference. Vertical black dashed lines connect data points to their corresponding statistical significance of comparisons at the top of the plot. (C) RNA Scope in-situ hybridization was performed on E16.5 wild-type matched littermate and *Zic3*-null spinal cords, showing qualitatively normal dILA neuron family location and numbers. For each genotype, a column of images showing a full spinal cord section and a column from a magnified region containing example dIL family subtypes are shown, with the field of view of the second column derived from the region within the box drawn with dashed white lines. White arrows indicate examples of dIL family subtypes. Other solid and dashed white lines indicate approximate anatomical boundaries of the spinal cord.
Numbers: Spinal cord sections from the same E16.5 embryos were used for both spatial transcriptomics and in-situ hybridization: n=3 wild-type, n=3 *Zic3*-null and n=3 *Zic1/4*^{DKO} with an additional five wild-type and *Zic3*-null embryos examined for in-situ hybridization alone (n=8 wild-type, n=8 *Zic3*-null). For spatial transcriptomics, numbers of E16.5 wild-type brachial neurons are noted in Figure S9, downsampled to 13,356 neurons of which 6,216 were classified as dIL. Additionally, cell coordinates were derived from two whole sections and one hemisection (N=2.5 sections) among n=3 replicate Zic3-null E16.5 spinal cords. From these sections 13,197 cells were identified as neurons and plotted, of which 6,753 were annotated as dIL neurons.
Statistics: (B) Two-way ANOVA (neuron subtype x genotype). For each neuron subtype in dILA2, Tukey's multiple comparisons test was performed for each pair of genotypes, the result of which is indicated by asterisks above the plot. **p<0.01, ***p<0.001
Scale bars: 250 µm, or 100 µm when magnified from boxes.
Abbreviations: ANOVA – Analysis of variance

Figure S18: Absence of *Zic1* and Zic4 systematically alters dIL subtype composition across families

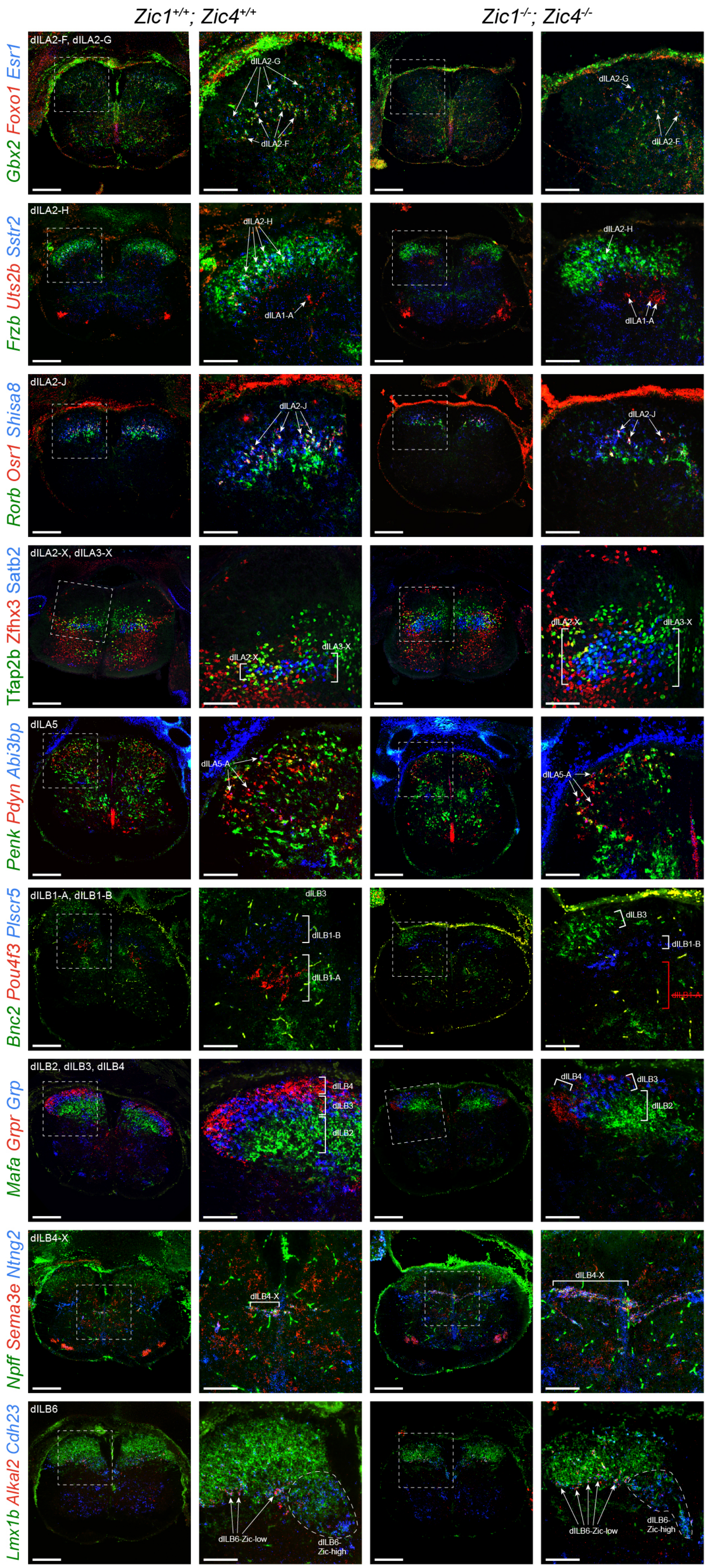

Figure S18: Absence of *Zic1* and *Zic4* systematically alters dIL subtype composition across families, related to Figure 5.

Images of immunohistochemistry and RNA Scope in-situ hybridization of selected dILA and dILB dorsal horn neuron types (indicated in the top left of each row) are examined in E16.5 spinal cords from wild-type (columns 1 and 2) or *Zic1/4*^{DKO} embryos (columns 3 and 4). For each genotype, a low magnification images (columns 1 and 3) and a high magnification image derived from boxes marked by dashed white lines in low magnification images (columns 2 and 4) are shown. White arrows or brackets indicate examples of dIL family subtypes. Other solid and dashed white lines indicate approximate anatomical boundaries of the spinal cord. Red text or brackets indicates depletion of neuron types in DKO spinal cords.
Numbers: Images obtained from representative n=3 wild-type and n=3 *Zic1/4*^{DKO} embryos.
Scale bars: 250 µm for columns 1 and 3; 100 µm for columns 2 and 4.
Abbreviations: DKO – double knock-out

Figure S19: Laminar position of dIL family subtypes in *Zic1/4*^{DKO} spinal cords

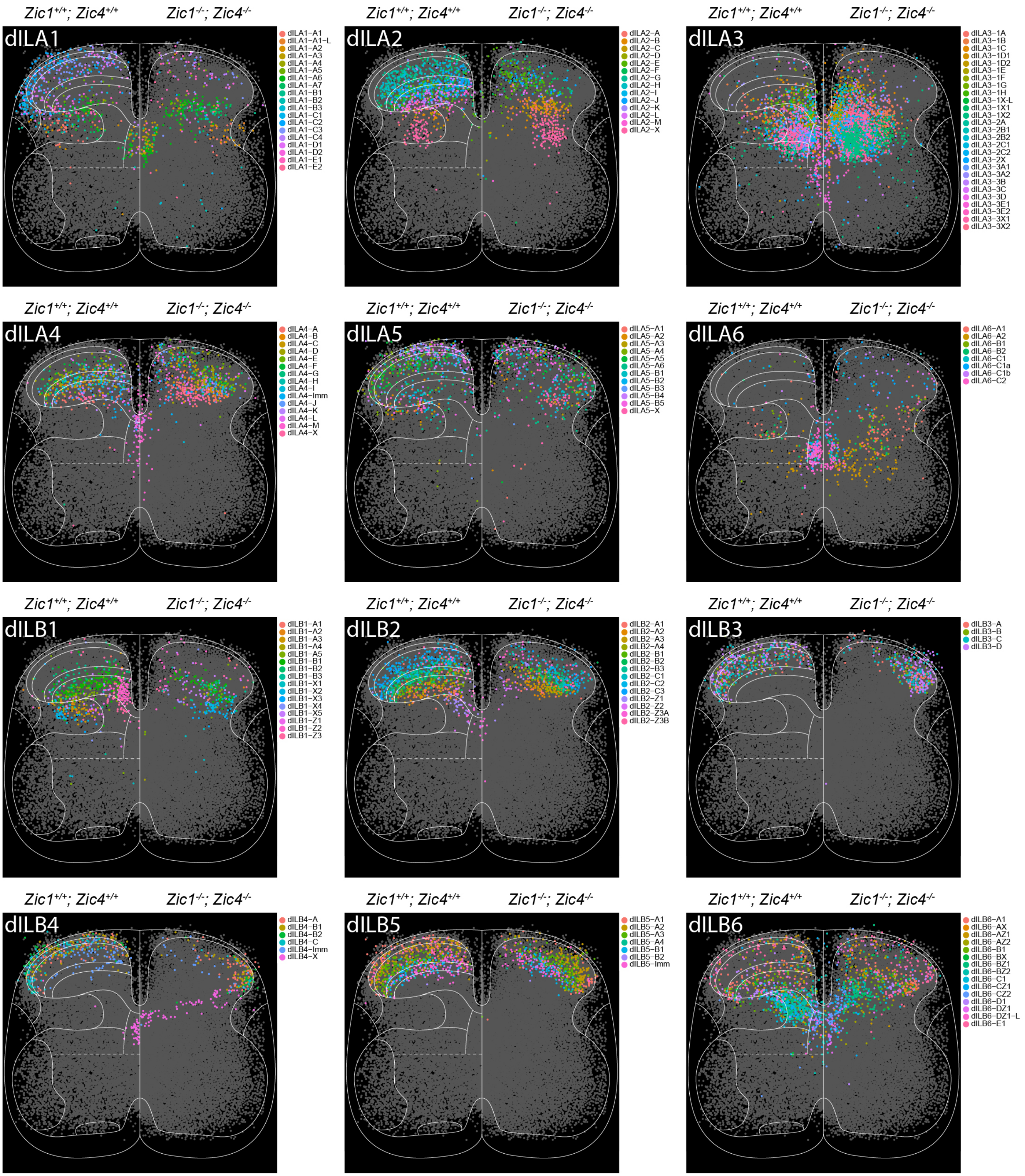

Figure S19: Laminar position of dIL family subtypes in *Zic1/4*^{DKO} spinal cords, related to Figure 5.

Scatter plots depict the coordinates of brachial wild-type and *Zic1/4*^{DKO} E16.5 neurons examined in the Xenium spatial transcriptomics assay, sorted into each of six dILA and six dILB families, and colored by subtype annotation. As described previously, wild-type cells are displayed on the left and null cells on the right of each diagram, and wild-type cells were downsampled to match the number of null cells. Cells not belonging to the indicated dIL family are plotted in gray. Solid and dashed white lines indicate approximate anatomical boundaries of the spinal cord. Numbers: Spatial transcriptomics from E16.5 wild-type embryo brachial segments was previously described (Fig. S9). For E16.5 *Zic1/4*^{DKO} brachial segments, cell coordinates were derived from three whole sections and one hemisection (N=3.5 sections) among n=3 replicate spinal cords. From these sections, 17,505 cells were identified as neurons, and of which 7,376 were dIL neurons. E16.5 brachial data was downsampled to 17,347 neurons of which 8,852 were dIL neurons to match.

Table S1: Single-cell RNA-sequencing sample statistics, related to Figure 1

Sample	Sample index	Cells retrieved	Mean reads per cell	Mean genes per cell	nFeature_RNA >	%percent.mt <	Neurons / sample	Age	Segment	Notes
14A	SI-GA-G1	12,634	41,083	2,678	500	5	2731	E14.5	Cervical	None
14B	SI-GA-G2	12,363	41,183	2,918	500	5	3336	E14.5	Cervical	None
14C	SI-GA-G3	9,576	46,151	2,359	500	5	1751	E14.5	Cervical	None
14D	SI-GA-G4	7,945	48,082	2,448	500	5	2493	E14.5	Cervical	None
14E	SI-GA-G5	13,317	34,404	1,594	500	5	2376	E14.5	Cervical	None
14F	SI-GA-G6	7,507	51,770	3,713	500	5	2563	E14.5	Cervical	None
16A	SI-GA-F7	13,350	45,507	3,059	500	5	3124	E16.5	Cervical	None
16B	SI-GA-F8	Excluded	Excluded	Excluded	Excluded	Excluded	Excluded	E16.5	Cervical	Poor emulsion; excluded
16C	SI-GA-G7	7,759	49,781	2,796	500	5	1045	E16.5	Cervical	None
16D	SI-GA-G8	8,245	50,412	2,836	500	5	1037	E16.5	Cervical	None
16E	SI-GA-G9	12,730	43,743	2,463	500	5	3093	E16.5	Cervical	None
16F	SI-GA-G10	8,399	49,602	3,203	500	5	2752	E16.5	Cervical	None
16G	SI-GA-H1	15,775	43,041	2,973	500	5	2769	E16.5	Cervical	None
16H	SI-GA-H2	14,174	41,088	2,806	500	5	2664	E16.5	Cervical	None
16LA	SI-GA-H3	11,834	48,483	3,718	200	8	4023	E16.5	Lumbosacral	None
16LB	SI-GA-H4	35,257	15,364	1,266	200	8	2944	E16.5	Lumbosacral	None
16LC	SI-GA-H5	7,802	66,608	4,403	200	8	3822	E16.5	Lumbosacral	None
16LD	SI-GA-H6	8,509	36,892	3,267	200	8	3893	E16.5	Lumbosacral	None
16LE	SI-GA-H7	7,383	106,417	5,059	**3000**	8	2615	E16.5	Lumbosacral	Minor emulsion failure; filters set high
16LF	SI-GA-H8	8,258	65,626	4,334	200	8	3428	E16.5	Lumbosacral	None
16LG	SI-TT-A1	8,858	57,587	5,112	200	8	3846	E16.5	Lumbosacral	None
16LH	SI-TT-A2	9,583	63,766	4,999	200	8	3673	E16.5	Lumbosacral	None
16TA	SI-GA-E12	993	178,101	730	500	5	32	E16.5	Thoracic	None
16TB	SI-GA-F10	10,666	51,835	4,624	**2500**	5	4550	E16.5	Thoracic	Minor emulsion failure; filters set high
16TC	SI-GA-F11	16,619	31,028	3,876	500	5	7114	E16.5	Thoracic	None
16TD	SI-GA-F12	15,928	32,305	3,790	500	5	6994	E16.5	Thoracic	None
16TE	SI-TT-A3	29,398	28,327	1,623	500	5	4218	E16.5	Thoracic	None
16TF	SI-TT-A4	6,598	111,576	6,382	500	5	4659	E16.5	Thoracic	None
16TG	SI-TT-A5	7,840	94,765	5,769	200	8	3168	E16.5	Thoracic	None
16TH	SI-TT-A6	8,122	76,001	5,651	200	8	3335	E16.5	Thoracic	None

Table S3: Neuron family statistics

neuron types	frequency	percent composition	Mean genes per cell	Mean silhouette score	Mean LISI score
CSF-CN	811	0.862325621	4884.347719	0.564226197	1.068983446
dI1	3098	3.294062606	5593.61685	0.02699042	1.11943434
dI2	3783	4.022414086	5764.700767	-0.055296513	1.103688505
dI3	1604	1.705512079	5677.580424	0.208083592	1.119244123
dI4	819	0.870831916	5523.943834	0.22369405	1.316595595
dI5	709	0.753870364	6353.574048	0.19264621	1.127166402
dI6	2617	2.78262164	5539.52159	-0.013385167	1.246705571
dILA1	3668	3.900136101	4889.60578	0.086811215	1.034098062
dILA2	8516	9.054950663	4559.639854	0.11291544	1.050834646
dILA3	8695	9.245279006	5023.275216	0.0328348	1.053900842
dILA4	4998	5.314307588	4691.958183	0.203464403	1.051976747
dILA5	5052	5.371725077	4250.411322	0.30324228	1.041783033
dILA6	4632	4.925144607	4335.858592	0.215672939	1.089141818
dILB1	2667	2.835785982	5618.602925	0.200086619	1.06214202
dILB2	6304	6.702960191	4299.443687	0.327612298	1.020879822
dILB3	4044	4.29993195	4275.0591	0.444091959	1.029691992
dILB4	1733	1.84267608	4914.615118	0.219520476	1.1210689
dILB5	4851	5.158004423	4677.446506	0.363075049	1.04413541
dILB6	6284	6.681694454	4478.111871	0.069308565	1.062557291
HB9-Int	78	0.082936373	5981.525641	0.427726905	1.108151671
hindbrain	290	0.308353181	3927.024138	0.111834411	Not calculated
Immature	5037	5.355775774	4094.726424	0.020971014	Not calculated
MN	794	0.844249745	6991.423174	0.273319801	1.018233824
other	108	0.114834978	6290.324074	0.228359563	Not calculated
v0c	233	0.247745832	6480.321888	0.119235925	1.279123907
v0d	1410	1.499234433	5945.839716	0.010501009	1.388347551
v0v	1171	1.245108881	5848.221178	-0.001023449	1.535806909
v1	3785	4.02454066	5796.785205	0.011365893	1.163589215
v2a	2011	2.13826982	5434.530085	0.060594438	1.081866375
v2b	2169	2.306269139	5348.930844	0.019703151	1.08873846
v3	2077	2.208446751	5778.061146	0.006636804	1.199208707

Table S5: Centroid-centroid distance between Russ et al. (2021) and Roome et al. annotations, related to Figure 1

	CSF-cN	MN	Npy	Rorb	Ml	Cdh3	Pdyn	Megf11	Maf	Cpne4	Prkcg	Reln	Rreb1	Sox5	ME/Lmx1b	ME	VE	Chat	Vl
CSF-CN	8.54960379	52.5809004	49.0890971	49.9099976	47.9810616	49.7906775	49.9441726	49.1898147	49.6647114	51.5704406	49.4072513	51.7448462	49.0895174	49.5589128	47.8428771	47.4912426	48.5379813	49.6881691	48.4940819
MN	45.6336675	0	29.083821	31.0961545	26.5862455	31.2063677	32.2539551	27.5117056	30.4893623	33.5496639	30.3856428	33.8213392	28.1150185	29.9260339	26.0733221	23.6063927	23.5469024	25.5438632	24.1396341
dILA1	41.4278342	28.1518935	3.03756802	16.6036074	11.6965996	18.9396917	18.8140188	16.3470503	19.4929894	23.4741737	19.2561145	23.5763896	16.6199657	18.7494493	15.339512	14.5522368	14.2541599	16.6624515	14.5889035
dILA2	42.8673465	31.4292082	17.069349	2.22983405	15.9406834	16.3928109	20.2651738	20.9738123	20.9584552	24.2443113	20.8772348	24.9578517	20.2944981	21.1821831	19.879464	19.7579267	20.1002213	20.8377523	20.2487574
dILA3	41.0010805	27.2487874	14.3823935	16.0288325	5.6890549	15.5805554	18.6576728	16.2418981	18.9309054	23.5543941	18.8918378	24.0777774	15.9608446	18.6061017	13.8779885	12.4081075	12.1708268	15.0881691	12.1143217
dILA4	43.4270087	32.670671	21.0761392	16.6831867	18.9618715	3.65751389	20.1305363	23.6214957	22.5959814	26.3355931	22.9877163	27.4852563	22.7171356	23.393549	21.8660161	21.8761961	22.7003962	21.6832822	22.4654949
dILA5	42.9815725	32.8653077	18.8128243	19.7135077	17.7834826	18.9240998	2.35754052	23.0024697	23.1321124	26.0460336	22.828247	25.8049896	20.4891794	21.8234554	20.2145343	20.6806401	22.230639	20.7189203	22.5654456
dILA6	40.8575553	32.555468	20.9045313	22.8174862	14.9850966	22.3134626	19.5684024	23.0590764	22.6760426	26.0678447	21.9919795	26.416491	20.2866062	21.8938187	16.9750667	17.6262615	21.1706092	21.918081	21.3039067
dILB1	42.3304096	27.8503936	18.5530342	21.5465752	16.6628092	22.8976937	23.6986743	2.34983414	18.7527987	23.9541791	19.9043915	24.7257858	16.6331449	19.4580231	14.2415595	15.3764243	13.9699977	19.8726	16.3073982
dILB2	43.9060451	33.1727388	23.0856271	23.2520794	21.6710158	24.1059758	25.3517808	21.1791207	6.19161868	7.15464416	6.66832826	20.5248389	20.2461056	19.729063	20.4722375	21.744041	22.6383438	24.3746754	23.7609459
dILB3	46.7725827	36.8772577	27.0925551	27.4174727	26.8500941	29.3621919	28.4874781	27.1697667	24.0527426	20.0516973	22.5064483	4.48024751	22.4541439	22.2575189	25.5466143	26.7929781	27.7516952	29.6477029	29.0519126
dILB4	43.2390029	30.3662369	19.9232165	21.7882655	18.5767295	23.3000096	22.2527684	18.4490102	19.7031556	22.0616829	19.0308362	17.9112992	5.55431904	11.9134629	16.9363697	17.8854592	18.6065928	21.9096938	20.5249007
dILB5	45.2262139	34.2887648	24.4709358	25.0430995	23.621684	26.3186448	25.7665531	23.6584326	21.9305946	23.7605892	20.7184871	22.2478756	17.1638005	7.25047143	22.3402134	23.3193416	24.2457105	26.2577336	25.7388231
dILB6	40.4916931	29.2773639	18.2218195	20.5234967	14.32653	20.7778323	20.1555211	15.5711024	17.5326779	21.8453234	16.9713189	22.4701789	14.4532121	17.0941907	5.53962418	13.5753769	16.2884234	19.1076745	18.2335338
dI1	41.3683879	25.2202284	17.6408715	20.8730154	13.3448383	21.8861411	22.8581881	14.7491419	19.8275715	24.1028237	19.3199232	24.2273689	15.8259057	18.5211658	12.1763547	8.93288252	5.619466	16.3504193	10.8321896
dI2	40.8270286	23.8874358	17.1178247	19.900647	11.5750405	20.3567478	21.6152401	14.0585278	18.7253908	22.9115313	18.2093587	23.6969689	14.1806433	17.6736562	11.1279262	6.65450624	5.04561491	15.4138643	8.02154616
dI3	41.3567615	22.4587432	17.5506941	21.0550357	13.2133432	22.0235029	23.075783	13.5014897	19.699662	24.1424178	19.5193595	24.6768688	16.388057	18.9168352	12.41921	8.5286062	3.93806516	15.8791	8.51346167
dI4	41.364224	25.0127455	14.4457223	18.8225094	10.1127405	19.9024957	21.8846504	15.504688	20.1977712	24.5345501	20.2253492	25.1312675	17.0142917	19.349999	14.3089547	11.0162338	7.19281492	13.3341636	4.89350445
dI5	42.0223606	21.9428681	18.0507349	22.0033635	15.5737376	22.472631	23.6783375	13.0692666	19.7310424	23.6609687	19.8968223	24.2270961	15.2916283	18.4569489	12.8401798	10.9503783	8.66366776	16.611516	12.2780805
dI6	41.3419646	24.8064699	17.2511452	20.2596013	11.797873	20.3689391	21.488667	17.2393958	20.4214289	24.5674294	20.3864027	25.4604542	18.0395305	20.2298897	15.0059919	11.7342916	10.5974566	6.43586969	7.89471402
v0d	40.9225096	23.9825438	17.3568584	20.5201042	10.7431926	20.5252061	22.1007619	16.1772148	20.176944	24.6636571	20.3394326	25.5734546	17.8055322	19.9994169	14.0693718	9.65166097	7.60933101	12.1094188	2.80294167
v0v	40.9977822	22.9172206	17.9575462	20.6857638	11.4880778	20.9591261	22.2906587	14.8031106	19.3591246	23.9973066	19.3304994	24.7116783	16.1524291	18.5726627	12.4208298	6.68369809	4.54182698	14.9032256	7.02559577
v0c	42.8492789	17.2930549	20.8264336	24.4815515	18.4649136	24.7085655	25.965537	19.0557532	23.6763828	27.4552963	23.5206497	27.7074123	19.7765503	22.2044209	17.3497484	13.2442541	12.78016	18.1283168	14.3132827
v1	41.4495143	24.6437634	17.4431941	20.623394	11.6798014	20.9235829	22.7600166	16.4401612	20.7782021	25.1836195	20.814922	26.1541412	18.0085129	20.3635709	14.7018496	10.0296641	7.11404339	15.1498682	3.24019325
v2a	42.4290214	26.0009364	20.7998076	24.0061984	17.1877034	24.2370078	25.1333495	19.2436798	22.4845085	26.5896956	22.2474517	26.9052849	19.8199615	21.897907	16.7880065	11.1623001	12.4330551	19.2974954	14.9216036
v2b	36.072427	25.4448519	17.6722473	20.616754	12.4721576	20.8310576	22.4912051	16.9082173	20.8260616	25.1036658	20.6317984	25.7440999	18.367253	20.372484	15.0397816	11.3504358	10.2588866	12.4752545	7.5745812
v3	40.4249155	23.5586361	18.5803565	21.5570879	12.7844508	21.9085529	22.8988005	15.8837827	20.0807913	24.7506824	19.9490066	25.0859743	16.9831207	19.2218854	13.3258481	7.19756389	7.13657987	16.5420739	10.6530484
HB9-Int	41.0942085	19.0725445	19.1981772	23.4572315	17.1425745	24.5152073	25.0056821	17.7012354	21.9220702	25.9452947	21.1423882	26.1689017	18.4900977	21.07249	16.4578536	10.963924	10.3436505	18.4686042	12.9102759

Table S9: Xenium gene panel, related to Figure 3

Genes	Ensembl_ID	Num_Probes	Codewords	Annotation	Panel
2600014E21R	ENSMUSG00	8	NA	NA	XED3D3
4933411E02R	ENSMUSG00	8	NA	NA	XED3D3
Abi3bp	ENSMUSG00	8	NA	NA	XED3D3
Alkal2	ENSMUSG00	8	NA	NA	XED3D3
Barhl1	ENSMUSG00	8	NA	NA	XED3D3
Bhlhe23	ENSMUSG00	8	NA	NA	XED3D3
Bnc2	ENSMUSG00	8	NA	NA	XED3D3
C1ql1	ENSMUSG00	6	NA	NA	XED3D3
C1ql3	ENSMUSG00	8	NA	NA	XED3D3
Calcr	ENSMUSG00	8	NA	NA	XED3D3
Cdh23	ENSMUSG00	8	NA	NA	XED3D3
Dmbx1	ENSMUSG00	8	NA	NA	XED3D3
Dmrt3	ENSMUSG00	8	NA	NA	XED3D3
Drd1	ENSMUSG00	8	NA	NA	XED3D3
En1	ENSMUSG00	7	NA	NA	XED3D3
Esr1	ENSMUSG00	8	NA	NA	XED3D3
Etv1	ENSMUSG00	8	NA	NA	XED3D3
Evx1	ENSMUSG00	8	NA	NA	XED3D3
Foxa2	ENSMUSG00	8	NA	NA	XED3D3
Foxb1	ENSMUSG00	8	NA	NA	XED3D3
Foxd3	ENSMUSG00	1	NA	NA	XED3D3
Foxo1	ENSMUSG00	8	NA	NA	XED3D3
Gal	ENSMUSG00	6	NA	NA	XED3D3
Gata3	ENSMUSG00	8	NA	NA	XED3D3
Gbx1	ENSMUSG00	8	NA	NA	XED3D3
Gbx2	ENSMUSG00	8	NA	NA	XED3D3
Gm6213	ENSMUSG00	8	NA	NA	XED3D3
Grp	ENSMUSG00	6	NA	NA	XED3D3
Grpr	ENSMUSG00	8	NA	NA	XED3D3
Il33	ENSMUSG00	8	NA	NA	XED3D3
Irx2	ENSMUSG00	8	NA	NA	XED3D3
Irx4	ENSMUSG00	8	NA	NA	XED3D3
Isl1	ENSMUSG00	8	NA	NA	XED3D3
Islr2	ENSMUSG00	8	NA	NA	XED3D3
Krt71	ENSMUSG00	8	NA	NA	XED3D3
Lbx1	ENSMUSG00	7	NA	NA	XED3D3
Lhx1	ENSMUSG00	8	NA	NA	XED3D3
Lhx2	ENSMUSG00	8	NA	NA	XED3D3
Lhx9	ENSMUSG00	8	NA	NA	XED3D3
Lmx1b	ENSMUSG00	8	NA	NA	XED3D3
Lpl	ENSMUSG00	8	NA	NA	XED3D3
Maf	ENSMUSG00	8	NA	NA	XED3D3
Mafa	ENSMUSG00	3	NA	NA	XED3D3
Mafb	ENSMUSG00	8	NA	NA	XED3D3
Mcoln3	ENSMUSG00	8	NA	NA	XED3D3
Mnx1	ENSMUSG00	8	NA	NA	XED3D3
Neurod2	ENSMUSG00	5	NA	NA	XED3D3
Neurog2	ENSMUSG00	8	NA	NA	XED3D3
Nfib	ENSMUSG00	8	NA	NA	XED3D3
Nkx2-2	ENSMUSG00	7	NA	NA	XED3D3
Nms	ENSMUSG00	8	NA	NA	XED3D3
Nmu	ENSMUSG00	6	NA	NA	XED3D3
Nos1	ENSMUSG00	8	NA	NA	XED3D3
Npas1	ENSMUSG00	4	NA	NA	XED3D3
Nr4a2	ENSMUSG00	8	NA	NA	XED3D3
Nr5a2	ENSMUSG00	8	NA	NA	XED3D3
Ntng2	ENSMUSG00	8	NA	NA	XED3D3
Nxph2	ENSMUSG00	8	NA	NA	XED3D3
Olig3	ENSMUSG00	8	NA	NA	XED3D3
Onecut1	ENSMUSG00	8	NA	NA	XED3D3
Osr1	ENSMUSG00	8	NA	NA	XED3D3
Otp	ENSMUSG00	8	NA	NA	XED3D3
Pax2	ENSMUSG00	8	NA	NA	XED3D3
Pax5	ENSMUSG00	8	NA	NA	XED3D3
Phox2a	ENSMUSG00	4	NA	NA	XED3D3
Pitx2	ENSMUSG00	8	NA	NA	XED3D3
Pkd2l1	ENSMUSG00	8	NA	NA	XED3D3
Plscr5	ENSMUSG00	8	NA	NA	XED3D3
Pou4f1	ENSMUSG00	8	NA	NA	XED3D3
Pou4f2	ENSMUSG00	8	NA	NA	XED3D3
Pou4f3	ENSMUSG00	8	NA	NA	XED3D3
Pou6f2	ENSMUSG00	6	NA	NA	XED3D3
Pth2r	ENSMUSG00	8	NA	NA	XED3D3
Rasgrp1	ENSMUSG00	8	NA	NA	XED3D3
Rxrg	ENSMUSG00	8	NA	NA	XED3D3
Shox2	ENSMUSG00	8	NA	NA	XED3D3
Sim1	ENSMUSG00	8	NA	NA	XED3D3
Skor2	ENSMUSG00	8	NA	NA	XED3D3
Slc18a3	ENSMUSG00	7	NA	NA	XED3D3
Slc32a1	ENSMUSG00	7	NA	NA	XED3D3
Sox1	ENSMUSG00	8	NA	NA	XED3D3
Sox6	ENSMUSG00	8	NA	NA	XED3D3
Sp8	ENSMUSG00	8	NA	NA	XED3D3
Sp9	ENSMUSG00	8	NA	NA	XED3D3
Syt10	ENSMUSG00	8	NA	NA	XED3D3
Tac1	ENSMUSG00	8	NA	NA	XED3D3
Tac2	ENSMUSG00	5	NA	NA	XED3D3
Tacr3	ENSMUSG00	8	NA	NA	XED3D3
Tfap2a	ENSMUSG00	8	NA	NA	XED3D3
Tfap2b	ENSMUSG00	8	NA	NA	XED3D3
Tnfaip8	ENSMUSG00	8	NA	NA	XED3D3
Tox2	ENSMUSG00	8	NA	NA	XED3D3
Vsx2	ENSMUSG00	8	NA	NA	XED3D3
Wt1	ENSMUSG00	8	NA	NA	XED3D3
Zfhx3	ENSMUSG00	8	NA	NA	XED3D3
Zic1	ENSMUSG00	8	NA	NA	XED3D3
Zic2	ENSMUSG00	8	NA	NA	XED3D3
Zic3	ENSMUSG00	8	NA	NA	XED3D3
Zic4	ENSMUSG00	8	NA	NA	XED3D3
Zic5	ENSMUSG00	8	NA	NA	XED3D3
